# The Spectroscopic Characterization of Halogenated Pollutants through the Interplay between Theory and Experiment: Application to R1122

**DOI:** 10.3390/molecules27030748

**Published:** 2022-01-24

**Authors:** Andrea Pietropolli Charmet, Giorgia Ceselin, Paolo Stoppa, Nicola Tasinato

**Affiliations:** 1 Dipartimento di Scienze Molecolari e Nanosistemi, Università Ca’ Foscari Venezia, Via Torino 155, I-30172 Mestre, Italy; stoppa@unive.it; 2 Scuola Normale Superiore, Piazza dei Cavalieri 7, I-56126 Pisa, Italy; giorgia.ceselin@sns.it

**Keywords:** quantum chemical calculations, ro-vibrational spectroscopy, environmental chemistry, IR spectroscopy

## Abstract

In the last decade, halogenated ethenes have seen an increasing interest for different applications; in particular, in refrigeration, air-conditioning and heat pumping. At the same time, their adverse effects as atmospheric pollutants require environmental monitoring, especially by remote sensing spectroscopic techniques. For this purpose, an accurate characterization of the spectroscopic fingerprint—in particular, those of relevance for rotational–vibrational spectroscopy—of the target molecules is strongly needed. This work provides an integrated computational–theoretical investigation on R1122 (2-Chloro-1,1-difluoro-ethylene, ClHC=CF${}_{2}$), a compound widely employed as a key intermediate in different chemical processes. State-of-the-art quantum chemical calculations relying on CCSD(T)-based composite schemes and hybrid CCSD(T)/DFT approaches are used to obtain an accurate prediction of the structural, rotational and vibrational spectroscopic properties. In addition, the equilibrium geometry is obtained by exploiting the semi-experimental method. The theoretical predictions are used to guide the analysis of the experimentally recorded gas-phase infrared spectrum, which is assigned in the 400–6500 cm${}^{-1}$ region. Furthermore, absorption cross sections are accurately determined over the same spectral range. Finally, by using the obtained spectroscopic data, a first estimate of the global warming potential of R1122 vibrational spectra is obtained.

## 1. Introduction

Halogenated ethenes are the shortest members among the family of halogenated olefins, a class of organic compounds that has received growing attention from the scientific community, especially in the last decade. In fact, some of them have been proposed as potential and environmentally tolerable alternatives to chlorofluorocarbons (CFCs) in refrigeration, air-conditioning and heat pumping applications. Containing at least one C=C double bond, they possess a reactive site that makes their scavenging from the atmosphere much more efficient. Thus, many studies have focused on the kinetics and mechanisms that may be involved in the atmospheric removal processes of these compounds, and so their reactions with OH radicals, as well as the ones with O(${}^{3}$P), have been investigated (see, for example [1,2,3,4,5,6,7] and references therein). However, it should be noted that some halogenated ethenes are considered to be potential threats to human health (see, for example [8,9,10] and references therein), so the assessing of their presence in real-time is clearly desirable, both for experimentally studying their atmospheric chemistry as well as for quantifying their concentrations. The recent advances in high-resolution infrared techniques make them very efficient in monitoring and detecting gaseous pollutants in real-time and with a very high sensitivity [11,12,13], provided that accurate spectroscopic data are available [14]. Generally, these data are obtained by the ro-vibrational and line-shape analysis of high-resolution spectra, which are often affected by several resonances [15]; ab initio calculations are nowadays able to yield accurate predictions for both anharmonic and Coriolis couplings, thus greatly assisting the whole process.

In addition, halogenated ethenes represent well-suited probes for characterizing the complex balance between van der Waals forces and steric effects that rules intermolecular interactions. Thus, many works have reported on the experimental and theoretical investigations carried out on the heterodimers between halogenated ethenes and noble gases [16,17,18], acids [19] or other different molecules [20,21].

Finally, the size of halogenated ethenes is small enough to make them appropriate test molecules to benchmark the computational predictions obtained at different levels of theory, from the state-of-the-art wavefunction-based methods, such as the coupled cluster with single and double excitations augmented by a perturbational estimate of the effects of connected triple excitations, CCSD(T) [22], to the less computationally demanding ones rooted in density functional theory (DFT). Within this framework, several investigations (see, for example, refs. [23,24,25,26,27,28,29] and references therein) have been carried out to disentagle the anharmonic interactions in their vibrational spectra and to assess the quality of the predicted dipole moment surfaces against the spectroscopic experimental data.

Among halogenated ethenes, 2-Chloro-1,1-difluoro-ethylene (R1122, ClHC=CF${}_{2}$) is currently employed as the key intermediate [30] in the production of different relevant compounds, such as fluorosurfactants, fluorine-containing textile finishing agents, organic silicon fluorine modified resins and other fine-chemical-containing fluorine.

Its microwave spectrum was first investigated by Jenkins et al. [31] in the 19–25 GHz region; almost ten years later, Stone and Flygare [32] extended the analysis in the range of 9.9–24.1 GHz to determine the off-diagonal terms of the ${}^{35}$Cl quadrupole coupling tensor. Subsequently, Leal et al. [33] analyzed the range of 12–225 GHz, thus obtaining a set of accurate rotational spectroscopic parameters for both the ${}^{35}$Cl and ${}^{37}$Cl species. Finally, Leung et al. [34], by using a combination of broadband chirped pulse and Balle–Flygare cavity Fourier transform microwave methods, derived the rotational and quartic centrifugal distortion constants also for the different deuterated and ${}^{13}$C isotopologues, as well as the corresponding chlorine nuclear quadrupole coupling tensor. In the same work, the gas-phase structure of the complex between R1122 and argon was investigated as well. Very recently, the structure of the heterodimer involving acetylene has also been analyzed [35]. On the other hand, concerning the infrared (IR) spectral region, to the best of our knowledge, the only data available in the literature are the ones reported by Nielsen et al. almost seventy years ago [36], where the proposed assignment of its absorption features was carried out mainly by correlating them to those previously assigned for CH${}_{2}$=CF${}_{2}$ and CH${}_{2}$=CCl${}_{2}$. In addition, there is no information about its absorption cross-section values or its atmospheric lifetime, both of which are mandatory to assess its global warming potential (GWP). Regardless, for the latter, Wuebbles et al. [37] reported the data of some similar unsaturated halogenated hydrocarbons, and they have maximum lifetime values in the range of 10–30 days.

In the present work, we carried out an integrated experimental–computational investigation on the structural and ro-vibrational spectroscopic properties of R1122, whose results can be used to guide further high-resolution analyses, as well as to improve the modeling of its atmospheric behaviour. A detailed analysis of the vibrational spectra of this molecule, comprehensive of an accurate determination of the corresponding absorption cross-section data up to 6500 cm${}^{-1}$, was coupled with high-level quantum chemical calculations performed at different levels of theory. Finally, the accurate values of the integrated band intensities allowed us to also estimate the radiative forcing (RF) and the corresponding GWP of R1122.

## 2. Materials and Methods

### 2.1. Computational Method

To accurately characterize the molecular structure and spectroscopic properties of ClHC=CF${}_{2}$ and to assist the vibrational analysis of experimentally recorded IR spectra, quantum chemical computations were carried out at different levels of theory in order to properly treat both electronic and nuclear problems. The equilibrium structure and the harmonic force field were computed by using the CCSD(T) method in conjunction with medium-to-large correlation-consistent cc-pV*n*Z basis sets with *n* = T, Q and 5 [38,39,40]. In order to reduce the one- and N-electron errors, different composite schemes were applied. In particular, the equilibrium structure was computed according to both the geometry gradient scheme [41] and the cheap composite scheme (ChS) [42]. Both of them consider the extrapolation to the complete basis set (CBS) limit and account for core correlation effects, but the former is formally justified by exploiting the additivity rule to build the gradient used in the geometry optimization, whereas the ChS assumes that the additivity relation can be applied directly on geometrical parameters. On the basis of the available data, both methods are expected to predict bond lengths and valence angles with an accuracy within 2 mÅ and 0.1−0.2°, respectively [43,44,45,46,47]. The ChS was also used for the computation of harmonic frequencies of vibration, as well as for obtaining quartic centrifugal distortion constants and nuclear quadrupolar coupling constants due to the presence of the Cl nucleus. Within the ChS, the estimate of the target property $p^{ChS}$ (*p* standing for structural parameters, quartic centrifugal distortion constants, nuclear quadrupolar coupling constants or harmonic vibrational frequencies) is obtained, adding on top of the CCSD(T)/cc-pVTZ ansatz corrections that account for the CBS extrapolation and core–valence correlation evaluated using the second-order Møller–Plesset (MP2) [48] perturbation theory.

Vibrational anharmonic contributions to the computed harmonic properties were evaluated through resorting to density functional theory (DFT). According to the recent literature, the hybrid B3LYP [49,50] and PW6B95 [51] functionals, in conjunction with a polarized double-ζ basis set, as well as the double-hybrids B2PLYP [52] and rev-DSDPBEP86 [53] joined with a triple-ζ basis set, can be recommended for the purpose in view of their good performance in the prediction of structural and ro-vibrational spectroscopic properties [54,55,56,57]. Specifically, the calendar basis sets [58] jul-cc-pVDZ and jun-cc-pVTZ were used in conjunction with the PW6B95 and double-hybrid functionals, respectively, whereas the SNSD [59] basis set was employed in B3LYP calculations. At all levels of theory considered, geometry optimizations were first carried out, followed by evaluation of analytical Hessians. Cubic and semidiagonal quartic force constants and second- and third-order derivatives of the dipole moment were obtained through numerical differentiation of analytical Hessian matrices, and first-order derivatives of the dipole moment surface, respectively. Double- and triple-ζ basis sets were supplemented by an additional set of *d* functions on the Cl atom in order to improve the accuracy of the results [55,60]. The relevant spectroscopic parameters were derived in the framework of vibrational perturbation theory to second-order (VPT2) [61,62,63] by using the computed equilibrium geometries, harmonic properties and anharmonic force constants. Coupled cluster computations were performed by using the CFOUR software [64], whereas MP2 and DFT calculations were carried out employing the Gaussian16 suite of programs [65], which was also adopted for applying VPT2 through its built-in generalized VPT2 engine [66,67]. The total energy distribution (TED) analysis of each normal mode was performed by using the INTDER2005 program [68].

### 2.2. Experimental Details

The gas-phase medium resolution (from 1.0 cm${}^{-1}$ to 0.2 cm${}^{-1}$) IR spectra of R1122 were recorded in the range of 400–6500 cm${}^{-1}$ by employing a Bruker Vertex 70 FTIR instrument and using a double-walled stainless steel cell fitted with KBr windows and with an optical path length of 134.0 (±0.5) mm. For the vibrational analysis, the spectra were recorded at room temperature, 128 scans were averaged and the pressure of the gas varied in the range of 2.7–200 hPa. For the determination of the absorption cross sections, the spectra were obtained at constant temperature (298.0 ± 0.5 K), the pressure of the gas varied in the range of 2.7–112 hPa (N${}_{2}$ was always added to obtain an overall pressure of 101 kPa, thus minimizing the bias due to finite resolution and instrumental distortion; see [69,70]) and up to 256 interferograms were acquired in order to increase the signal-to-noise ratio. Additional details on the whole experimental procedure can be found in our previous works (see, for example, refs. [25,47] and references therein).

## 3. Results and Discussion

**Figure 1:**
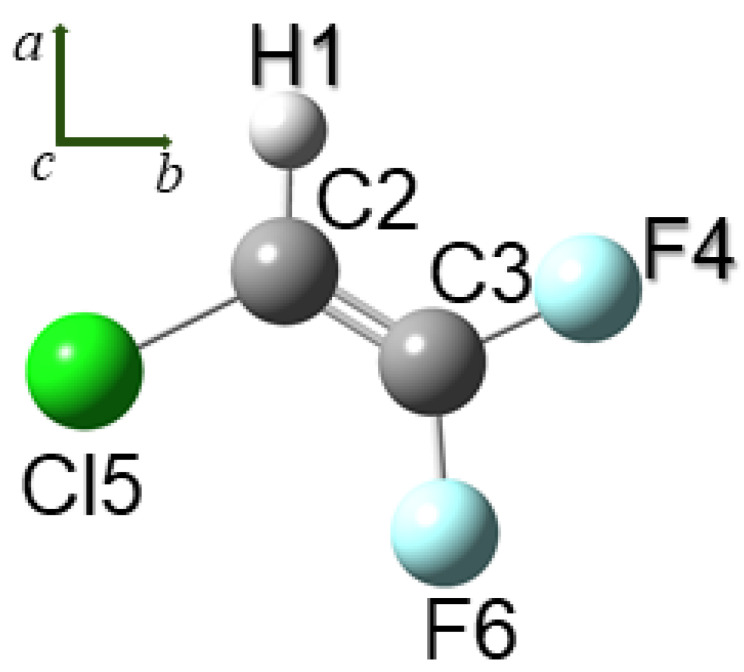
Structure of R1122 with atom labelling. The orientation of the principal axes of inertia is also shown.

R1122 is a planar near-prolate molecule, with asymmetry parameter κ≈−0.909, belonging to the $C_{S}$ symmetry point group. The *a* and *b* principal axes of inertia define the molecular plane, whereas the *c* axis is perpendicular to it, as illustrated in Figure 1. It possesses 12 normal modes of vibration that, in terms of the symmetry species, can be classified as $9A^{\prime }⨁3$$A^{\prime \prime }$; $A^{\prime }$ vibrations give rise to hybrid a/b bands, whereas vibrations of $A^{\prime \prime }$ symmetry produce *c*-type absorptions.

In the following equilibrium, geometries and rotational properties are discussed first, and then the attention is moved to the vibrational harmonic force field. Subsequently, anharmonic effects are introduced, and the issuing theoretical predictions used to drive the interpretation of the IR spectra are experimentally recorded. This subsection also deals with a description of the main absorption bands and the measurement of integrated absorption cross sections over the 400–6500 cm${}^{-1}$ spectral range.

### 3.1. Equilibrium Geometry and Ro-Vibrational Spectroscopy

The fundamental prerequisite for obtaining reliable predictions of spectroscopic parameters that are accurate enough to drive the interpretation of experimental spectra is an accurate molecular geometry, in particular, for what concerns rotational spectroscopy. Although the ground-state rotational constants of six isotopologues of ClHC=CF${}_{2}$ have been experimentally determined [34], to the best of our knowledge, only an effective vibrationally averaged structure (namely the so-called $r_{0}$ structure) has been proposed, with no attempt to derive the equilibrium geometry. In this respect, it is well known that the semi-experimental (SE) approach is the best method for obtaining accurate equilibrium structures for non-trivial (i.e., two- or three atom-) molecules in the gas-phase [71]. The method makes use of vibrational contributions evaluated theoretically, $\Delta B^{vib}$, to correct the experimentally derived rotational constants, usually those of the ground-vibrational state $B_{\alpha }^{0}$ (α=a,b,c being the principal axis of inertia), and obtain the so-called SE equilibrium rotational constants, $B_{\alpha }^{SE}$, of a set of isotopologues [72]:

(1) $$B_{\alpha }^{SE}=B_{\alpha }^{0}-\Delta B_{\alpha }^{vib}$$

For each isotopologue, vibrational corrections $\Delta B_{\alpha }^{vib}$ can be computed in the framework of VPT2 [61,62,63]. A non-linear least-squares fitting procedure is then employed to invert the set of SE equilibrium rotational constants to the molecular structural parameters, thus returning the SE equilibrium geometry.

To obtained the SE equilibrium geometry of R1122, the ground-state rotational constants measured for ${}^{35}$ClHC=CF${}_{2}$, ${}^{37}$ClHC=CF${}_{2}$, ClH${}^{13}$C=CF${}_{2}$, ClHC=${}^{13}$CF${}_{2}$, ${}^{35}$ClDC=CF${}_{2}$ and ${}^{37}$ClDC=CF${}_{2}$ [34] and the corresponding vibrational corrections computed at the rev-DSDPBEP86/jun-cc-pV(T+*d*)Z level of theory have been fed into the MSR software [73], which has been used for structural refinement. The lack of isotopic substitutions on F atoms, which prevents a reliable determination of the structural parameters involving them, can be overcome by using two strategies: the non-determinable parameters can be constrained to an accurate theoretical value or, alternatively, one can resort to the use of the predicate observations method, which uses estimates of structural parameters as additional input data [74]. In addition to being more flexible with respect to treating theoretical parameters as rigid constraints, it should lead to more precise results [75,76]. In the present work, this second method has been adopted with predicates for the C3−F4 and C3−F6 bond lengths and C2C3F4 and C2C3F6 angles taken from the CCSD(T)/CBS+CV gradient scheme results. The final fit converged to the SE equilibrium geometry reported in Table 1, with a root mean square deviation of $1.7\times 10^{-3}$ u Å${}^{2}$. All of the structural parameters are well determined, with maximum standard deviations of 1.1 mÅ for bond lengths and around 0.1° for angles, although, for the C3C2H1 angle, the 95% confidence interval represents a safer estimate of its accuracy.

Indeed, the same table also lists the equilibrium geometry of R1122 obtained from the CCSD(T)/CBS+CV and ChS composite methods and at the CCSD(T)/cc-pV5Z level of theory. The two composite schemes are in perfect agreement with the SE equilibrium geometry, with the largest deviation being 0.9 mÅ and 1.6 mÅ for bond lengths at the CCSD(T)/CBS+CV and ChS level, respectively, and within 0.1° for bond angles. The only exception is the C3C2H1 angle, which is 0.5° lower accordingly to the theoretical predictions. The equilibrium geometry obtained by using the large cc-pV5Z basis set is also in agreement with the SE structure, even though larger deviations than those for composite methods can be noted. A strikingly accurate equilibrium geometry is obtained by augmenting the PW6B95 and rev-DSDPBEP86 results through the nano-LEGO approach [76]. In fact, whereas for the bare functionals, differences as large as 6–7 mÅ are obtained, as can be seen in Table S1 of the Supplementary Materials (SM), the nano-LEGO procedure lowers the deviations to 1.2 mÅ, thus reaching the same accuracy as the CCSD(T)-based composite methods but at a far lower computational cost. Concerning valence angles, the deviations obtained for the C3C2Cl angle at the DFT level are somewhat larger than those stemming from CCSD(T)-based approaches, but this is due to the lack of the nano-LEGO parameterization for this angle. By comparing the SE geometrical parameters with the theoretical counterparts obtained by the different methods, it can be speculated that the SE value of the C3C2H1 angle may be overestimated by about 0.5°. A possible explanation may be related to the imprecision of the rotational constants experimentally determined for the deuterated species, that, in turn, affects the fitting procedure used for the structural refinement.

Moving from the equilibrium geometries, the corresponding equilibrium rotational constants have been derived and, then, by augmenting them through vibrational corrections at the DFT level, ground state rotational constants have been obtained. The rotational constants of ${}^{35}$ClHC=CF${}_{2}$ are compared to the available experimental results in Table 2 together with quartic centrifugal distortion parameters (data refer to the Watson’s A-reduction Hamiltonian in the $I^{r}$ representation) and nuclear quadrupolar coupling constants. The high accuracy obtained in the equilibrium geometry is mirrored in the predicted ground state rotational constants, which reproduce the experimental values with a mean absolute percentage error (MAPE) around 0.06% when the composite schemes are considered. The same accuracy is reached by the nano-LEGO corrected rev-DSDPBEP86 functional, which significantly improves the results delivered by the bare functional (MAPE =0.7%), as can be appreciated from Table S2 of the Supplementary Materials. A sensible improvement is also noted by comparing PW6B95+nano-LEGO (MAPE =0.2%) with the corresponding non-augmented counterpart (MAPE =0.7%), which, in any case, overshoots the accuracy of the rotational constants obtained at the B3LYP/SNSD level of theory (MAPE =2%, see Table S2 of Supplementary Materials). In passing, it is interesting to note that both the CCSD(T)/CBS+CV and ChS composite methods, and also the nano-LEGO augmented functionals, yield predictions that are more accurate than the computationally demanding CCSD(T)/cc-pV5Z level of theory, which reproduces the experimental outcomes with a MAPE of around 0.4%. Furthermore, by comparing the experimental data [33,34] with the theoretical counterparts, it is noted that the values obtained in ref. [33] appear to be more accurate than those in ref. [34], which is also coherent with their superior precision, probably because, in the former investigation, the effects of centrifugal distortion have been described up to terms depending on the sixth power of the angular momentum operators, whereas, in the latter, the rotational Hamiltonian has been truncated at the quartic terms. A good agreement can also be reported between the experimental and theoretical quartic centrifugal distortion constants obtained from the ChS, which, on average, achieve the same accuracy (MAPE =1.3%) as the CCSD(T)/cc-pV5Z computations, but with a significantly lower computational cost. The only notable difference between the two is the maximum deviation, which amounts to −4.6% and 1.8% for the ChS and CCSD(T)/cc-pV5Z, respectively. The rev-DSDPBEP86 and PW6B95 show slightly larger errors, the MAPE being around 2% and 4% in line with previous benchmark studies [54,55,57].

Sextic centrifugal distortion constants have been computed, and are listed in Table 3, where the comparison with the available experimental data [33] is also given, again referring to the Watson’s A-reduction Hamiltonian in the $I^{r}$ representation. As can be seen, the theoretical results obtained at the different levels of theory employed are in good overall agreement, with only the PW6B95 seeming to yield slightly larger values for the $\Phi_{J}$ and $\Phi_{JK}$ centrifugal distortion parameters. Comparing the computed vales of $\Phi_{JK}$, $\Phi_{KJ}$ and $\phi_{JK}$ with the experimental counterparts, a general good agreement can be noted, even though, according to the expected accuracy of the calculations [46,77], the relative deviations between 10 and 15% suggest that the rotational spectra of this molecule may deserve additional investigations, with the aim of extending the analysis toward higher rotational quantum numbers (*J* in particular). This should lead to the determination of the missing sextic centrifugal distortion parameters, which, in turn, can also affect the values of the remaining ones. In addition, or alternatively, a fit constraining the not-yet-determined parameters to the theoretical values should be performed. This may avoid the determinable sextic centrifugal distortion constants being biased in the attempt to account for the centrifugal distortions effects described by the missing parameters. In this regard, the values obtained from the HYB-1 and HYB-2 force fields are suggested for the purpose: the former has been obtained by using the CCSD(T)/CBS+CV geometry, ChS harmonic frequencies (see next subsection) and rev-DSDPBEP86/jun-cc-pV(T+*d*)Z cubic force constants; the latter has been derived by mixing the CCSD(T)/cc-pV5Z geometry and harmonic force field with CCSD(T)/cc-pVTZ cubic force constants. For the sake of completeness, the rotational spectroscopic parameters of the ${}^{37}$ClHC=CF${}_{2}$, ${}^{35}$ClHC=${}^{13}$CF${}_{2}$, ${}^{35}$ClH${}^{13}$C=CF${}_{2}$, ${}^{35}$ClDC=CF${}_{2}$ and ${}^{37}$ClDC=CF${}_{2}$ isotopic species, obtained at different levels of theory, can be found in Tables S3–S7 of the Supplementary Materials.

Before concluding this subsection, the $\alpha_{k}^{\beta }$ (*k* and β=a,b,c representing the vibrational normal mode and principal axis of inertia, respectively) ro-vibrational interaction constants and the $\zeta_{kl}^{\alpha }$ Coriolis coupling parameters (in absolute value), which are relevant for further ro-vibrational spectroscopic high-resolution spectroscopic investigations, are listed in Table 4 and Table 5, respectively. Concerning the Coriolis resonance, it should be recalled that R1122 vibrational levels belonging to the same symmetry species can interact through *c*-type Coriolis interactions, whereas levels of different symmetries can be coupled by both *a*- and *b*-type Coriolis interactions. By using the $\alpha_{k}^{\beta }$ values, vibrational contributions to rotational constants have been worked out and employed to correct the equilibrium rotational constants corresponding to the SE structure, thus obtaining the rotational constants of the singly excited fundamental vibrational levels reported in Table 6.

### 3.2. Harmonic Force Field

Harmonic wavenumbers of ClHC=CF${}_{2}$ fundamental vibrations are listed in Table 7, together with the description of the vibrational normal modes based on total energy distribution (TED) values (%) obtained in terms of the internal coordinates defined in the lower part of the same table. For each normal mode, the TED analysis has been carried out in terms of a set of internal coordinates and using the quadratic force constants obtained at the fc-CCSD(T) level of theory. Intensities computed within the double-harmonic approximation are also reported, with the ChS harmonic intensity of the normal mode *i*, $I_{i}^{ChS}$ computed according to the following expression:

(2) $$I_{i}^{ChS}=I_{i}^{CCSD(T)/VTZ}+\Delta I_{i}^{MP2/(T-Q)}+\Delta I_{i}^{MP2/CV}$$

where the first term on the r.h.s. is the harmonic intensity at the CCSD(T)/cc-pV(T+*d*)Z level, whereas the second and the third terms account for the enlargement of the basis set and the contribution from the correlation of core electrons, respectively. The former is obtained as the difference between MP2 values computed with the cc-pV(Q+*d*)Z and cc-pV(T+*d*)Z basis sets, whereas the latter is the difference between intensities calculated at the MP2/cc-pwCVTZ level by correlating all and only valence electrons. While representing an empirical approximation, the reliability of this approach has been shown to provide reliable predictions [59]. As can be seen, both the frequencies and intensities obtained at the CCSD(T)/cc-pV5Z and ChS levels are in very good agreement. Indeed, the composite approach, while mostly relying on MP2 computations, reproduces the CCSD(T)/cc-pV5Z results with an average accuracy of only 2 cm${}^{-1}$ and a maximum difference of 5 cm${}^{-1}$ reported for the $\omega_{3}$ and $\omega_{10}$ vibrations. Concerning harmonic intensities, the two methods agree on average within 0.7 km mol${}^{-1}$, with the largest difference of 3.8 km mol${}^{-1}$ observed for the $\omega_{4}$ vibration, that, given the strong intensity of this vibration, in relative terms, corresponds to only the 3%. Table 7 also lists the harmonic wavenumbers and intensities of R1122 computed at the rev-DSDPBEP86/jun-cc-pV(T+*d*)Z and PW6B95/jul-cc-pV(D+*d*)Z levels of theory, whereas results from B2PLYP/jun-cc-pV(T+*d*)Z and B3LYP/SNSD computations can be found in Tables S8 and S9 of the Supplementary Materials, respectively. Both rev-DSDPBEP86 and PW6B95 confirm their reliability for computing vibrational properties [55]; in particular, CCSD(T)/cc-pV5Z harmonic frequencies are reproduced with a MAD of only 4.2 cm${}^{-1}$ by the rev-DSDPBEP86 double-hybrid functional and 12.8 cm${}^{-1}$ by the PW6B95 hybrid functional, whereas, for harmonic intensities, the MADs amount to 2.1 and 4.4 km mol${}^{-1}$, respectively. As can be seen by looking at the results reported in Table 7, the TEDs of the $\omega_{5}$, $\omega_{6}$, $\omega_{7}$ and $\omega_{8}$ normal modes show significant contributions from bending and/or stretching involving the chlorine atom; therefore, the corresponding instances of ${}^{35/37}$Cl isotopologue splitting should be visible in the experimental spectra.

### 3.3. Vibrational Spectroscopy beyond the Double-Harmonic Approximation

In order to obtain theoretical predictions that are usable for the quantitative interpretation of experimentally recorded spectra, both mechanical and electrical anharmonicity need to be considered in the calculations. For the purpose, fundamental vibrational frequencies and IR intensities computed beyond the double-harmonic approximation are reported in Table 8, together with the experimentally measured wavenumbers (for a graphical comparison between the theory and experiment, see Figure S1 of the Supplementary Materials), and in Table 9, respectively. While a detailed interpretation of the experimental spectra is deferred to the next subsection, here, a comparison among the theoretical outcomes obtained at the different levels of theory is given, pointing out both their accuracy with respect to the experiment and the most important aspects required for the spectral interpretation. Table 8 reports the predictions according to four different hybrid force fields: in the CC5Z:rDSD and CC5Z:PW6 ones, the harmonic properties from CCSD(T)/cc-pV5Z computations have been mixed with anharmonic contributions evaluated by using the rev-DSDPBEP86 and PW6B95 functionals, respectively; ChS:rDSD and ChS:PW6 are the similar counterparts, but are obtained using harmonic frequencies and IR intesities at the ChS level. In addition, the table also collects the predictions from full rev-DSDPBEP86/jun-cc-pV(T+*d*)Z and PW6B95/jul-cc-pV(D+*d*)Z computations, whereas B2PLYP and B3LYP results can be found in the Supplementary Tables S8 and S9, respectively. The different hybrid force fields yield very similar results for the fundamental vibrational frequencies and, in general, they agree within a few wavenumbers. This is particularly important for the $\nu_{9}$, $\nu_{11}$ and $\nu_{12}$ fundamentals, for which accurate and reliable theoretical predictions become important for the assignment of overtones and combination bands, due to the lack of experimental observations. Indeed, the $\nu_{9}$ vibration is predicted to occur at quite low wavenumbers, in a region that is difficult to access experimentally, whereas the $\nu_{11}$ and $\nu_{12}$ vibrations, both having an intensity of around 0.5 km mol${}^{-1}$ at the anharmonic level, produce absorptions that are too weak to be directly detected, even if the $\nu_{12}$ frequency has been estimated from the measurement of difference bands (vide infra). Both the highest levels of theory used in the present work, i.e., CC5Z:rDSD and ChS:DSD, place $\nu_{9}$, $\nu_{11}$ and $\nu_{12}$ at 195, 580 and 235 cm${}^{-1}$, respectively, and these values have been used for assisting the spectral interpretation (vide infra). Before moving to the interpretation of the R1122 vibrational signatures, it is interesting to note that the $\nu_{3}$ vibration is involved in a Fermi resonance of type 2 with the $\nu_{10}+\nu_{11}$ combination, which is predicted to be particularly strong at the ChS:rDSD level of theory due to the closeness of their deperturbed vibrational energies. In fact, while this interaction is of a weaker magnitude at the other levels of theory employed, it is described by the following matrix by the ChS:rDSD hybrid force field:

$v_{3}=1$$v_{10}=v_{11}=1$$v_{3}=1$1333.1−7.2$v_{10}=v_{11}=1$−7.21334.4
whose eigenvalues and eigenvectors are, respectively:
23261341$0.52|\nu_{3}\rangle$$0.48|\nu_{10}+\nu_{11}\rangle$$0.48|\nu_{10}+\nu_{11}\rangle$$0.52|\nu_{3}\rangle$

As a matter of fact, the $v_{3}=1$ and $v_{10}=v_{11}=1$ levels are mixed to the same extent in the perturbed states, thus making an assignment in terms of unperturbed level labels meaningless. Given this caveat, the interpretation of the experimental spectrum remains unaffected and independent of the model hybrid calculation.

### 3.4. Interpreting Experiments: Vibrational Analysis and Absorption Cross Sections

**Figure 2:**
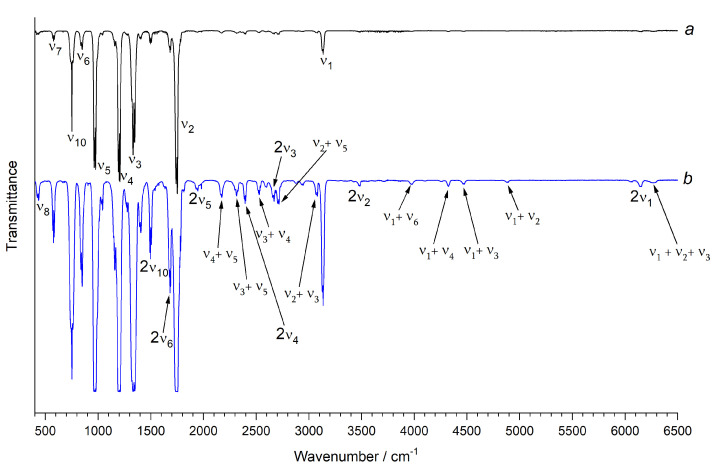
Survey infrared spectra of R1122 in the 400–6500 cm${}^{-1}$ spectral region. Resolution = 1.0 cm${}^{-1}$, KBr windows, path length = 13.4 cm, room temperature; pressure = 345 Pa (trace a, in black) and 88.77 hPa (trace b, in blue). Only some representative bands are labeled.

The vibrational analysis was performed on the gas-phase spectra measured in the range of 400–6500 cm${}^{-1}$. The first step was the assignment of all of the strongest fundamentals, which was carried out on the spectra obtained at lower pressures; then, the weaker absorption features (mainly due to overtone and combination bands) were identified in the spectra obtained at higher pressures. A survey spectrum of the overall region investigated is reported in Figure 2. Table 8 lists all of the assigned fundamentals, together with the corresponding predicted values obtained at different levels of theory; in the same way, Table 9 reports the corresponding predicted anharmonic intensities. Finally, Table 10 comprises all of the vibrational assignments carried out in the present work, together with the corresponding predicted values, whereas the anharmonic constants $x_{ij}$ derived from the assigned bands are reported in Table S12 of the Supplementary Materials, where, for completeness, the full list of theoretical values obtained from the CC5Z:rDSD hybrid force field is also given. For the sake of comparison, the measured fundamental frequencies of R1122 are juxtaposed with those of similar halogenated ethenes (H${}_{2}$C=CHCl [78], H${}_{2}$C=CHF [79] *cis-* and *trans-* ClHC=CHF [80], ClFC=CF${}_{2}$ [25]) in Table S13 of the Supplementary Materials.

On the basis of quantum chemical calculations, the $\nu_{9}$ and $\nu_{12}$ fundamentals are expected to be around 195 and 235 cm${}^{-1}$, respectively. Even though, for the latter one, the transition frequency has been experimentally confirmed by the observation of the $\nu_{5}$–$\nu_{12}$ difference band, the recording of their spectra may require a dedicated investigation through, e.g., the use of synchrotron radiation facilities [81,82]. Furthermore, at room temperature, the two lowest-lying vibrational levels $v_{9}=1$ and $v_{12}=1$ present a relative population of approximately 39% and 32%, respectively, with respect to the ground vibrational state. For this reason, the absorptions due to hot bands contribute significantly to the room temperature IR spectra, even if, at the resolutions employed in the present work, they usually overlap and are covered by the stronger envelopes of the cold bands. If, on the one hand, their assignment can be attempted by recording spectra at higher resolutions (e.g., 0.1 cm${}^{-1}$ or better), on the other, the spectral congestion stemming from hot band absorptions can make high-resolution investigations challenging, thus requiring cold spectra [83].

#### 3.4.1. 400–800 cm${}^{-1}$ Spectral Region

This spectral region is dominated by the strong absorption (with a predicted intensity in the range of 34–36 km mol${}^{-1}$; see Table 9) due to the $\nu_{10}$ band ($A^{\prime \prime }$ symmetry), located at 751.1 cm${}^{-1}$, being in good agreement with the calculated anharmonic values (obtained at different levels of theory) listed in Table 8. In addition, there are also two weaker fundamentals of $A^{\prime }$ symmetry ($\nu_{7}$ and $\nu_{8}$) with a computed intensity (at the CC5Z:rDSD level of theory) of 3.13 and 1.43 km mol${}^{-1}$, respectively; concerning the position of $\nu_{8}$, there is a very good agreement between its experimental value (at 431.8 cm${}^{-1}$) and the predicted ones, which are in the range of 430–432 cm${}^{-1}$. Moving to higher wavenumbers, it is worthwhile to note that the stronger $\nu_{7}$, located at 578.0 cm${}^{-1}$, completely obscures the signals coming from the very weak $\nu_{11}$, predicted in the range of 580–594 cm${}^{-1}$ and with a computed intensity lower than 1 km mol${}^{-1}$. Focusing on the position of $\nu_{7}$, all of the theoretical methods listed in Table 8 (with the exception of the PW6B95 hybrid) led to predicted values having an absolute error that is not greater than one wavenumber. Besides, it is worthwhile to note that, even if on the basis of the TED analysis (see Table 7), we could expect to see the instances of the ${}^{35/37}$Cl isotopologue splitting for both of these two fundamentals (i.e., $\nu_{7}$ and $\nu_{8}$), only for $\nu_{7}$ are these features clearly visible in the spectra (the signals are located at 578.0 and 577.4 cm${}^{-1}$, respectively), whereas, in the case of $\nu_{8}$, they were not discernible (due to the predominant B-type envelope of this band). The agreement between the experimentally determined and the predicted positions for all of the bands assigned in this spectral region can be considered as very remarkable (the MAD is only 1.9 and 1.4 cm${}^{-1}$ at the CC5Z:rDSD and ChS:rDSD levels of theory, respectively; see the data listed in Table 10). Finally, it is worthwhile to note that, using the $\nu_{5}$–$\nu_{12}$ located at 736.2 cm${}^{-1}$, it is possible to predict the position of $\nu_{12}$, thus obtaining a value of 235.3 cm${}^{-1}$, which is in very good agreement with the ab initio data of 235 and 236 cm${}^{-1}$ yielded by the CC5Z:rDSD and ChS:rDSD levels of theory, respectively (see Table 8).

#### 3.4.2. 800–1800 cm${}^{-1}$ Spectral Region

As expected by the analysis of the data reported in Table 9, in this spectral region, the key features (which characterize the spectra measured at lower pressures) are the strong absorptions due to the $\nu_{5}$ (at 971.5 cm${}^{-1}$), $\nu_{4}$ (at 1200 cm${}^{-1}$), $\nu_{3}$ (at 1341.7 cm${}^{-1}$) and $\nu_{2}$ (at 1747.5 cm${}^{-1}$) fundamentals (all having $A^{\prime }$ symmetry), which have computed intensities, at the CC5Z:rDSD level of theory, in the range of 84–142 km mol${}^{-1}$. Located at lower wavenumbers, there is the much weaker $\nu_{6}$ band (centered at 844.9 cm${}^{-1}$, with a predicted intensity lower than 9 km mol${}^{-1}$). The $\nu_{5}$ and $\nu_{6}$ fundamentals show distinct absorption features (located at 971.5/970.2 and 844.9/841.8 cm${}^{-1}$, respectively) due to the presence of both the ${}^{35/37}$Cl isotopologues, thus being in line with the predictions made on the basis of the corresponding TED analysis (as reported in Table 7). Looking at the theoretical data, what is remarkable is the agreement between the CC5Z:rDSD predictions and the corresponding experimental values of these bands (the greatest error is less than two wavenumbers). The spectra recorded at increasing pressures allowed for the identification of several signals assigned to two-quanta combinations (for example, $\nu_{7}$ + $\nu_{8}$, $\nu_{6}$ + $\nu_{9}$, $\nu_{5}$ + $\nu_{7}$, $\nu_{4}$ + $\nu_{7}$ and so on), as well as to overtone bands (2$\nu_{7}$, 2$\nu_{6}$ and 2$\nu_{10}$). Even in this spectral region, the comparison between the measured positions of the assigned bands (fundamentals and many two-quanta transitions) and the predicted values (listed in Table 10) points out the overall very good performance of the calculations carried out at the CC5Z:rDSD level of theory: the corresponding MAD is less than 2 cm${}^{-1}$, whereas the computed data at the ChS:rDSD level of theory has a MAD of 2.5 cm${}^{-1}$.

#### 3.4.3. 1800–3200 cm${}^{-1}$ Spectral Region

In the range of 1800–3200 cm${}^{-1}$, only the $\nu_{1}$ fundamental ($A^{\prime }$ symmetry, located at 3135.9 cm${}^{-1}$) is clearly visible in the spectra recorded at low pressures (as expected by looking at the corresponding calculated intensity, 12.07 km mol${}^{-1}$ at the CC5Z:rDSD level of theory; see Table 9). Increasing the sample pressure allowed us to identify and assign the signals coming from the 2$\nu_{5}$ (at 1939.8 cm${}^{-1}$), 2$\nu_{4}$ (at 2394.7 cm${}^{-1}$) and 2$\nu_{3}$ (at 2663.7 cm${}^{-1}$) overtones, as well as several absorptions due to binary combinations mainly involving $\nu_{2}$ or $\nu_{3}$ (such as $\nu_{2}$ + $\nu_{12}$, $\nu_{3}$ + $\nu_{5}$, $\nu_{2}$ + $\nu_{7}$, $\nu_{3}$ + $\nu_{4}$, $\nu_{2}$ + $\nu_{5}$, $\nu_{2}$ + $\nu_{3}$). Looking at the comparison between the experimental and the predicted values (as reported in Table 10) the overall agreement is still very good, with the MAD being around 3.5 cm${}^{-1}$ for both the CC5Z:rDSD and ChS:rDSD levels of theory.

#### 3.4.4. 3200–6500 cm${}^{-1}$ Spectral Region

The analysis of the signals falling in the last spectral region (3200–6500 cm${}^{-1}$) was carried out by using the spectra measured at a high sample pressure, and several two- and three-quanta combination bands (mainly involving $\nu_{1}$) were assigned. Besides them, the high-wavenumber side of this region is characterized by the 2$\nu_{1}$ overtone (at 6150.1 cm${}^{-1}$) and the nearby $\nu_{1}$ + $\nu_{2}$ + $\nu_{3}$ band. Due to the presence of absorptions involving only two- and (some) three-quanta transitions, this spectral region can be considered as more challenging for the theoretical predictions than the former ones; regardless, the overall agreement reached at the CC5Z:rDSD level of theory is very good, with the MAD being only 4.1 cm${}^{-1}$, whereas, in this spectral region, the ChS:rDSD predictions present a larger MAD of 9 cm${}^{-1}$. Despite this, the computed anharmonic frequencies and intensities are accurate enough to lead to an unambiguous assignment of the observed spectral features.

Using the positions of the fundamentals as benchmark data to assess the performances of the different anharmonic force fields employed in the present work, the results reported in Table 8 point out the excellent accuracy of CC5Z:rDSD; the MAD is only 1.4 cm${}^{-1}$, and all of the bands are predicted with errors that are generally within a few wavenumbers (the largest absolute deviation is smaller than 4 cm${}^{-1}$). For comparison, all of the other composite schemes yielded slightly larger MAD values, even if their predictions can still be considered as more than satisfactory; regardless, they all led to bigger deviations of up to around 8 cm${}^{-1}$ for some bands. The very remarkable accuracy offered by the CC5Z:rDSD method is further confirmed by taking into account the whole set of assigned transitions (thus including many overtone and combination bands, and, in some cases, up to three quanta; see Table 10); the overall MAD is less than 3 cm${}^{-1}$, and most of the deviations are generally lower than 6 cm${}^{-1}$. For comparison, the predictions obtained at the ChS:rDSD level of theory have an overall MAD of 4.4 cm${}^{-1}$.

#### 3.4.5. Absorption Cross Sections and Integrated Band Intensities

In the present work, the determination of the absorption cross section spectra of R1122 was carried out by using the medium resolution spectra and following the procedure described in detail elsewhere (see, for example, ref. [84] and references therein). Briefly speaking, the method is based on the least-squares fitting the point-by-point absorbance value, A($\tilde{\nu }$) measured at each wavenumber, $\tilde{\nu }$, versus the corresponding sample concentration, and always using N${}_{2}$ as an inert buffer gas. The slope that is thus obtained at each wavenumber, σ($\tilde{\nu }$), gives the absorbance cross section per molecule (cm${}^{2}$ molecule${}^{-1}$); in the same way, the point-by-point error estimate is also obtained (as a statistical uncertainty). It is worthwhile to note that this procedure avoids the distortion due to saturation effects and, at the same time, leads to a better signal-to-noise ratio for the weaker signals; besides, we demonstrated that it produces data in very good agreement with the ones yielded by the line-shape analyses carried out on high-resolution measurements (see, for example, refs. [85,86,87,88]).

**Figure 3:**
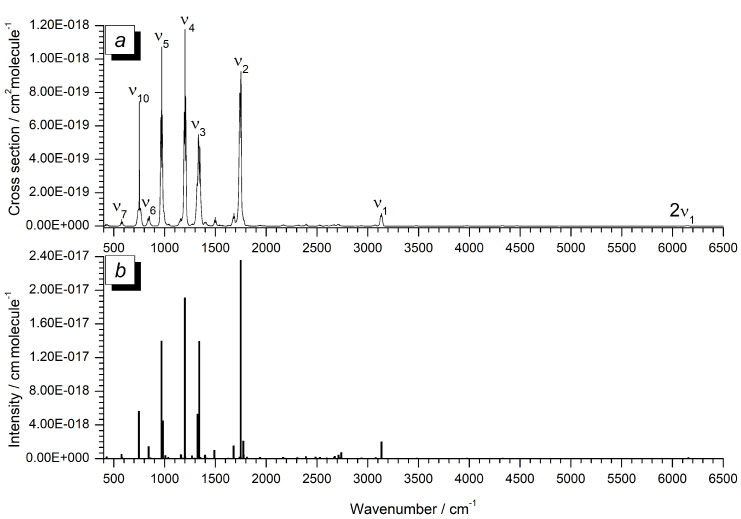
(**a**) Experimental cross-section spectrum of R1122 in the 400–6500 cm${}^{-1}$ spectral region. Resolution = 0.2 cm${}^{-1}$, KBr windows, path length = 13.4 cm, 298.0 ± 0.5 K. Only some representative bands are labeled. (**b**) Theoretical stick spectrum obtained at the hybrid CC5Z:rDSD level of theory over the same spectral range.

The cross-section spectrum of the overall region investigated is reported in Figure 3a, where it is also compared with the theoretical stick spectrum obtained from hybrid CC5Z:rDSD computations (panel b of the same Figure). As can be seen, the match between the measured and theoretical wavenumbers and relative intensities is very pleasant. In making the comparison, it should be stressed that, whereas the stick spectrum refers to anharmonic IR intensities, the experimental trace represents the cross section spectrum, which yields band intensities upon integrating over a given spectral interval, as explained above. Indeed, Table 11 lists the experimental integrated absorption cross sections (cm molecule${}^{-1}$) together with the corresponding theoretical predictions obtained at both the CC5Z:rDSD and ChS:rDSD levels of theory.

In the spectral range of 400–900 cm${}^{-1}$ the $\nu_{10}$ band is the most intense absorption; its measured integrated intensity, 5.531(34) × 10${}^{-18}$ cm molecule${}^{-1}$, clearly overcomes the other fundamentals falling in this region ($\nu_{6}$ has a value of 1.605(10) × 10${}^{-18}$ cm molecule${}^{-1}$, whereas $\nu_{7}$ and $\nu_{8}$ have cross-sections of 6.503(80) and 2.33(27) × 10${}^{-19}$ cm molecule${}^{-1}$, respectively).

The strongest absorptions (accounting for more than 68% of the overall integrated band intensities) are localized in the region of 900–1900 cm${}^{-1}$, and they are due to the $\nu_{2}$, $\nu_{3}$, $\nu_{4}$ and $\nu_{5}$ fundamentals, which is as expected since all of their descriptions involve a significant fraction of C-F stretchings (see the corresponding TED% in terms of internal coordinates reported in Table 7). Their individual integrated intensities range from 1.8 to 2.7 × 10${}^{-17}$ cm molecule${}^{-1}$, and their overall sum is equal to 8.50 × 10${}^{-17}$ cm molecule${}^{-1}$. At this point, it is worthwhile to note that the overall cross section of R1122 in the atmospheric window (900–1400 cm${}^{-1}$) can be considered as rather large (around 5.9 × 10${}^{-17}$ cm molecule${}^{-1}$), in line with that of other similar halogenated ethenes (for comparison, ClFC=CH${}_{2}$ has an integrated value of almost 4.5 × 10${}^{-17}$ cm molecule${}^{-1}$; see [23], whereas that of ClFC=CF${}_{2}$ is around 9.3 × 10${}^{-17}$ cm molecule${}^{-1}$, see [25]).

The $\nu_{1}$ fundamental, with an integrated intensity of almost 2.14 × 10${}^{-18}$ cm molecule${}^{-1}$, clearly dominates the region around 3000 cm${}^{-1}$. The other absorptions, falling at higher wavenumbers, i.e., in the range of 3440–6500 cm${}^{-1}$, are mainly due to overtone and combination bands, and their overall integrated cross section is around 4 × 10${}^{-19}$ cm molecule${}^{-1}$.

Concerning the comparison between the experimental data and the corresponding computed values, the data reported in Table 11 highlight the very good overall performance of both the CC5Z:rDSD and ChS:rDSD levels of theory in reproducing the most intense absorptions (i.e., the ones falling in the region of 900–1900 cm${}^{-1}$), with the average absolute errors being not greater than 6%. The predicted intensities for the weaker features (such as the combination and overtone bands) show larger deviations (in line with the trends seen in previous investigations; see, for example, ref. [84] and references therein), but the average absolute error, considering the whole data set of integrated cross-sections up to 6300 cm${}^{-1}$, is less than 3.5 × 10${}^{-19}$ cm molecule${}^{-1}$ (i.e., around 8% of the value of the overall integrated intensity).

By using the obtained cross section spectrum within the narrowband model of Ref. [89], the radiative forcing (RF) of R1122 has been estimated to be 0.098 W m${}^{2}$ ppbv${}^{-1}$. Even if we are aware that this approach is not well suited for short-lived molecules and therefore this determined value should be considered as just an estimate of the actual RF, we note that the data thus computed are in good agreement with the ones obtained using a more sophisticated model on a similar halogenated olefin (e.g., *trans*-1-chloro- 3,3,3-trifluoropropylene, see [37]). As pointed out in the introduction, for the atmospheric lifetime of R1122, a reasonable guess of the upper value should be in the range of 10–30 days (see also [90]), thus leading to an estimated GWP between 1.5 and 4.5 on a 100-year time horizon, and between 6 and 18 on a 20-year time horizon.

## 4. Conclusions

Spectroscopic remote sensing techniques are widely used to probe the Earth’s atmosphere, to retrieve its composition and to monitor the concentration profiles of a number of species; in particular, anthropogenic pollutants, which may have hazardous environmental effects or contribute to global climate change. In order to exploit the observational data, spectroscopic information needs to be accurately determined for the species of potential interest. This represent a huge and time-consuming task that, because of the difficulties in interpreting the experimentally recorded spectra, can be fruitfully achieved by coupling laboratory experiments with state-of-the-art quantum chemical simulations. In the present work, the integrated experimental–theoretical approach to the spectroscopic characterization of atmospheric pollutants has been presented, pointing out the accuracy requirements of quantum chemical calculations for the quantitative interpretation of experimental spectra and using the R1122 molecule as a case study. In particular, a comprehensive characterization of the structural and rotational/vibrational spectroscopic properties of R1122 has been performed. First, the equilibrium geometry has been derived by the semi-experimental approach, in which the ground-state rotational constants of a set of isotopologues have been corrected through vibrational contributions evaluated at the rev-DSDPBEP86/jun-cc-pV(T+*d*)Z level and used to refine the structural parameters in a non-linear least-squares procedure. The equilibrium geometry has also been theoretically derived by adopting different methods; in particular, CCSD(T)-based composite schemes, as well as DFT computations relying on the rev-DSDPBEP86 or PW6B95 functionals corrected by the recently proposed Nano-LEGO platform. The theoretical geometries have resulted in a very good agreement with the SE structure, with the deviations being around 1–2 mÅ and 0.2° for the bond lengths and angles, respectively. Next, the parameters relevant for rotational spectroscopy, i.e., ground state rotational constants, quartic- and sextic-centrifugal distortion constants and Cl-nuclear quadrupolar coupling constants, have then been derived for the different isotopologues of the molecules by means of approaches rooted in CCSD(T) and DFT methods, and have then been compared to the available experimental data. In this respect, the theoretical sextic centrifugal distortion parameters can be used to extend the knowledge of the R1122 rotational fingerprint and to drive the assignment toward high *J* values; for the isotopologues containing ${}^{37}$Cl, ${}^{13}$C and D atoms, sextic distortion parameters are here estimated for the first time. Finally, the vibrational spectroscopic properties have been accurately simulated by accounting for both mechanical and electrical anharmonicity in the framework of VPT2 applied to hybrid force fields, in which the harmonic properties derived from the CCSD(T)/cc-pV5Z or ChS computations have been mixed with anharmonic effects evaluated at the rev-DSDPBEP86/jun-cc-pV(T+*d*)Z level of theory. Moving from the simulated IR spectra, a complete analysis of the experimentally measured gas-phase IR spectra of R1122 in the range of 400–6500 cm${}^{-1}$ has been carried out. The vibrational features have been assigned in terms of fundamentals, overtones and combination bands up to three quanta, and the corresponding absorption cross-sections have been accurately determined over the same spectral range. Finally, the obtained vibrational spectroscopic data have been employed to obtain the first estimate of the R1122 radiative forcing and, from this, its global warming potential over the 20- and 100-year time horizon.

## Figures and Tables

**Table 1:** Semi-experimental (SE) and theoretical equilibrium geometry of ClHC=CF${}_{2}$${}^{a}$.

Parameter	SE ${}^{b}$	CCSD(T)/CBS+CV	ChS	CCSD(T)/V5Z	PW6-nL ${}^{c}$	rDSD-nL ${}^{d}$
*r*(C2−H1)	1.07479(34;79)	1.0753	1.0753	1.0763	1.0756	1.0748
*r*(C2=C3)	1.3236(11;27)	1.3226	1.3216	1.3259	1.3235	1.3234
*r*(C3−F4)	1.31421(88;207)	1.3135	1.3157	1.3156	1.3129	1.3140
*r*(C2−Cl5)	1.71000(71;169)	1.7099	1.7140	1.7152	1.7088	1.7087
*r*(C3−F6)	1.3073(12;27)	1.3068	1.3084	1.3090	1.3062	1.3071
α(C3C2H1)	120.89(8;20)	120.38	120.64	120.39	120.43	120.29
α(C2C3F4)	123.16(9;22)	123.16	123.29	123.15	123.30	123.19
α(C3C2Cl5)	121.567(41;97)	121.65	121.43	121.65	122.03	121.94
α(C2C3F6)	125.78(6;14)	125.71	125.72	125.72	125.62	125.46

*^a^* Bond lengths in Å, bond angles in deg. *^b^* Figures in parentheses are standard deviation and 95% confidence intervals in the units of the last significant digits. *^c^* PW6B95/jul-cc-pV(D + *d*)Z equilibrium geometry augmented through Nano-LEGO. *^d^* rev-DSDPBEP86/jun-cc-pV(T+*d*)Z equilibrium geometry augmented through Nano- LEGO.

**Table 2:** Theoretical rotational-, quartic centrifugal distortion- and nuclear quadrupolar coupling constants of ${}^{35}$ClHC=CF${}_{2}$ and comparison to experimental values ${}^{a}$.

	CCSD(T)/CBS+CV ${}^{b}$	ChS ${}^{c}$	CCSD(T)/V5Z ${}^{d}$	PW6-nL ^e^	rDSD-nL ${}^{f}$	Exp. ${}^{g}$	Exp. ${}^{h}$
$A_{0}$	10,718.563	10,723.797	10,680.042	10,741.852	10,712.614	10,710.7335(14)	10,710.73661(64)
$B_{0}$	2298.164	2298.203	2286.337	2292.253	2295.206	2297.18531(61)	2297.18720(14)
$C_{0}$	1891.014	1891.203	1881.791	1887.676	1888.822	1890.14572(36)	1890.14644(15)
MD%	−0.05	−0.07	0.40	0.02	0.05	-	-
MAD%	0.05	0.07	0.40	0.21	0.06	-	-
$\Delta_{J}$	n.a.	0.350	0.346	0.355	0.339	0.333(12)	0.348727(26)
$\Delta_{JK}$	n.a	4.26	4.13	4.44	4.077	3.95(11)	4.07532(51)
$\Delta_{K}$	n.a.	7.75	7.74	7.61	7.69	8.17(11)	7.8803(52)
$\delta_{J}$	n.a.	0.0596	0.0589	0.06042	0.05790	0.0588(11)	0.059845(8)
$\delta_{K}$	n.a.	2.60	2.574	2.733	2.544	2.418(97)	2.6008(14)
MD%	n.a.	−0.58	0.76	−2.67	2.12	-	-
MAD%	n.a.	1.39	1.33	4.04	2.14	-	-
$\chi_{aa}$	−54.3	−56.0	−55.3	−51.0	−51.8	−54.8923(48)	−54.81(8)
$\chi_{bb}$	17.7	18.3	18.0	17.7	17.4	18.2356(57)	18.18(4)
$\chi_{cc}$	36.1	37.2	36.7	33.3	34.3	36.6567(56)	36.63(6)
$|\chi_{ab}|$	45.7	46.4	46.3	43.0	43.5	47.02(13)	n.a.

*^a^* Rotational parameters within theWatson’s A-reduction Hamiltonian in the *I^r^* representation. Rotational- and nuclear quadrupolar coupling constants in MHZ; quartic centrifugal distortion constants in kHz. *^b^* Equilibrium rotational constants at CCSD(T)/CBS+CV level corrected through rev-DSDPBEP86/jun-cc-pV(T+*d*) vibrational contributions. Nuclear quadrupolar coupling constants at CCSD(T)/cc-pVQZ level augmented through rev- DSDPBEP86/jun-cc-pV(T+*d*) vibrational contributions. *^c^* Equilibrum rotational constants corresponding to the ChS geometry corrected through rev-DSDPBEP86/jun-cc-pV(T+*d*) vibrational contributions. Nuclear quadrupolar coupling constants from ChS augmented through rev-DSDPBEP86/jun-cc-pV(T+*d*) vibrational contributions. *^d^* Equilibrium rotational constants at CCSD(T)/cc-pV5Z level corrected through rev-DSDPBEP86/jun-cc-pV(T+*d*) vibrational contributions. *^e^* Equilibrum rotational constants from Nano-LEGO PW6B95 geometry corrected through PW6B95/jul-cc-pV(D + *d*)Z vibrational contributions. Centrifugal distortion- and nuclear quadrupolar coupling constants from the bare functional. *^f^* Equilibrum rotational constants from Nano-LEGO rev-DSDPBEP86 geometry corrected through rev-DSDPBEP86/jun-cc-pV(T+*d*) vibrational contributions. Centrifugal distortionand nuclear quadrupolar coupling constants from the bare functional. *^g^* From Ref. [34]. *^h^* From Ref. [33].

**Table 3:** Sextic centrifugal distortion constants (Hz) of ${}^{35}$ClHC=CF${}_{2}$${}^{a}$.

	HYB-1 ${}^{b}$	HYB-2 ${}^{c}$	CCSD(T)/VTZ	PW6	rDSD	Exp. ${}^{d}$
$\Phi_{J}$× 10${}^{5}$	6.26	6.65	6.47	7.63	6.03	n.a.
$\Phi_{JK}$× 10${}^{3}$	4.20	4.21	4.06	5.62	4.01	n.a.
$\Phi_{KJ}$	−0.0319	−0.0329	−0.0315	−0.0354	−0.0308	−0.0278(24)
$\Phi_{K}$	0.058	0.058	0.056	0.061	0.056	0.067(15)
$\phi_{J}$× 10${}^{5}$	1.80	1.90	1.85	2.00	1.73	n.a.
$\phi_{JK}$× 10${}^{3}$	2.00	2.03	1.96	2.65	1.92	2.25(39)
$\phi_{K}$	0.0909	0.0884	0.0870	0.1025	0.0876	n.a.

*^a^* Watson’s A-reduction Hamiltonian in the *I^r^* representation. *^b^* CCSD(T)/CBS+CV geometry, cheap harmonic frequencies and rev-DSDPBEP86/jun-cc-pV(T+*d*)Z cubic force constants. ^*c*^ Geometry and harmonic frequenciesat CCSD(T)/cc-pV5Z level and cubic force constants from CCSD(T)/cc-pVTZ computations. *^d^* From Ref. [33].

**Table 4:** $\alpha_{k}^{\beta }$ vibrational–rotational interaction constants (MHz) of ${}^{35}$ClHC=CF${}_{2}$.

Normal Mode	*a*	*b*	*c*
1	16.342	2.277	2.014
2	30.201	6.317	4.909
3	31.581	1.337	2.815
4	5.389	1.442	2.336
5	18.068	0.569	0.577
6	−10.680	6.059	5.114
7	0.399	−1.473	−0.048
8	−16.074	1.164	1.833
9	95.383	1.932	1.982
10	6.997	−1.148	−1.999
11	14.983	−0.176	−0.969
12	−76.684	−3.812	−2.423

**Table 5:** Relevant Coriolis coupling constants of ${}^{35}$ClHC=CF${}_{2}$.

*a*-Type Coriolis			*b*-Type Coriolis			*c*-Type Coriolis		
Mode *k*	Mode *l*	**|$\zeta_{kl}^{a}$|**	Mode *k*	Mode *l*	**|$\zeta_{kl}^{b}$|**	Mode *k*	Mode *l*	**|$\zeta_{kl}^{c}$|**
1	10	0.973	2	10	0.412	1	2	0.232
1	11	0.173	2	11	0.719	1	3	0.519
2	11	0.478	2	12	0.155	1	4	0.780
1	12	0.119	3	10	0.550	1	5	0.233
3	10	0.110	3	11	0.218	1	6	0.324
3	11	0.750	3	12	0.178	2	3	0.654
4	11	0.232	4	10	0.690	2	4	0.325
5	11	0.219	4	11	0.552	2	5	0.170
6	10	0.171	4	12	0.300	2	6	0.509
6	11	0.211	5	10	0.167	2	7	0.236
6	12	0.471	5	12	0.787	2	8	0.252
7	12	0.543	6	11	0.148	3	4	0.239
8	11	0.171	6	12	0.310	3	5	0.156
8	12	0.371	7	11	0.304	3	7	0.446
9	12	0.551	8	12	0.111	3	8	0.143
			9	11	0.120	4	5	0.128
			9	12	0.355	4	7	0.304
						4	8	0.309
						4	9	0.136
						5	6	0.473
						5	7	0.838
						5	8	0.159
						5	9	0.731
						6	7	0.504
						7	8	0.491
						7	9	0.165
						8	9	0.469

**Table 6:** Rotational constants (MHz) of the excited fundamental vibrational levels of ${}^{35}$ClHC=CF${}_{2}$.

Vibrational Levels	*A*	*B*	*C*
$v_{1}=1$	10,694.453	2294.943	1888.133
$v_{2}=1$	10,680.573	2290.896	1885.255
$v_{3}=1$	10,679.194	2295.873	1887.323
$v_{4}=1$	10,705.395	2295.783	1887.803
$v_{5}=1$	10,692.714	2296.652	1889.572
$v_{6}=1$	10,721.464	2291.166	1885.045
$v_{7}=1$	10,710.372	2298.691	1890.201
$v_{8}=1$	10,726.861	2296.053	1888.313
$v_{9}=1$	10,615.398	2295.273	1888.163
$v_{10}=1$	10,703.777	2298.361	1892.150
$v_{11}=1$	10,695.802	2297.402	1891.131
$v_{12}=1$	10,787.479	2301.029	1892.570

**Table 7:** TED, harmonic wavenumbers (cm${}^{-1}$) and intensities (km mol${}^{-1}$) of ${}^{35}$ClHC=CF${}_{2}$ normal modes of vibration.

		Chs		CCSD(T)/V5Z		PW6		rDSD	
Mode	TED%	ω	*I*	ω	*I*	ω	*I*	ω	*I*
$\omega_{1}$	$R_{1}$(99.5)	3265	14.34	3261	14.48	3286	17.60	3268	15.64
$\omega_{2}$	$R_{2}\left(72.6\right)-R_{5}\left(9.7\right)-R_{3}\left(7.4\right)$	1789	172.38	1786	173.84	1821	187.02	1789	185.47
$\omega_{3}$	$R_{5}\left(38.4\right)-R_{6}\left(29.8\right)-R_{3}\left(25.8\right)+R_{9}\left(4.6\right)$	1365	118.68	1370	120.52	1347	128.80	1362	114.04
$\omega_{4}$	$R_{6}\left(60.2\right)-R_{3}\left(32.3\right)$	1221	118.61	1224	122.48	1206	128.53	1225	134.28
$\omega_{5}$	$R_{5}\left(31.9\right)-R_{4}\left(23.3\right)+R_{3}\left(19.7\right)+R_{2}\left(13.3\right)$	991	113.90	991	113.88	991	129.69	993	119.75
$\omega_{6}$	$R_{4}\left(44.1\right)-R_{7}\left(17.0\right)+R_{5}\left(15.2\right)-R_{9}\left(11.3\right)$	858	8.23	857	8.54	858	10.26	856	9.05
$\omega_{7}$	$R_{8}\left(66.5\right)-R_{3}\left(11.0\right)-R_{7}\left(9.8\right)+R_{9}\left(6.8\right)$	584	3.04	583	3.20	575	2.78	583	2.96
$\omega_{8}$	$R_{9}\left(64.0\right)+R_{4}\left(25.1\right)+R_{7}\left(7.9\right)-R_{8}\left(6.8\right)$	437	1.51	436	1.53	434	1.57	437	1.39
$\omega_{9}$	$R_{7}\left(63.3\right)+R_{8}\left(27.4\right)-R_{9}\left(8.2\right)$	196	1.85	195	1.90	188	1.98	196	1.89
$\omega_{10}$	$R_{12}\left(82.3\right)+R_{11}\left(25.1\right)+R_{10}\left(-7.3\right)$	769	35.88	764	35.30	781	37.68	776	37.21
$\omega_{11}$	$R_{10}\left(112.0\right)+R_{12}\left(-14.5\right)$	592	0.89	591	0.89	604	0.36	606	0.52
$\omega_{12}$	$R_{11}\left(72.5\right)-R_{12}\left(32.2\right)-R_{10}\left(-4.7\right)$	238	0.59	238	0.62	241	0.70	240	0.54
**Internal coordinates definition**									
$R_{1}$ = C−H stretching					$R_{2}$ = C−C stretching				
$R_{3}$ = C−F4 stretching					$R_{4}$ = C−Cl stretching				
$R_{5}$ = C−F6 stretching					$R_{6}$ = C−C−H bending				
$R_{7}$ = C−C−Cl bending					$R_{8}$ = C−C−F6 bending				
$R_{9}$ = C−C−F4 bending					$R_{10}$ = F−C−C−F out of plane				
$R_{11}$ = H−C−C−Cl out of plane					$R_{12}$ = H−C−C−F4 torsion				

**Table 8:** Experimental and theoretical wavenumbers (cm${}^{-1}$) for ClHC=CF${}_{2}$ fundamental vibrations.

Mode	Exp.	CC5Z:rDSD ${}^{a}$	CC5Z:PW6 ${}^{b}$	ChS:rDSD ${}^{c}$	ChS:PW6 ${}^{d}$	rDSD	PW6
$\nu_{1}$	3135.9(3)	3139	3134	3139	3138	3143	3161
$\nu_{2}$	1747.5(1)	1749	1751	1751	1753	1752	1786
$\nu_{3}$	1341.7(3)	1342	1348	1341/1326 ^e^	1343	1328	1321
$\nu_{4}$	1200.7(1)	1200	1209	1196	1206	1201	1192
$\nu_{5}$	971.5(1)/970.2(1) ${}^{f}$	970	973	972/970 ${}^{f}$	975/973 ${}^{f}$	974/972 ${}^{f}$	975/973 ${}^{f}$
$\nu_{6}$	844.9(1)/841.8(5) ${}^{f}$	843	845	845/842 ${}^{f}$	847/844 ${}^{f}$	844/841 ${}^{f}$	846/844 ${}^{f}$
$\nu_{7}$	578.0(1)/577.4 ${}^{f}$	577	579	578/577 ${}^{f}$	579/579 ${}^{f}$	577/577 ${}^{f}$	570/569 ${}^{f}$
$\nu_{8}$	431.8	431	432	432/428 ${}^{f}$	433/429 ${}^{f}$	431/427 ${}^{f}$	430/426 ${}^{f}$
$\nu_{9}$	n.a.	195	202	195/193 ${}^{f}$	202/201 ${}^{f}$	195/194 ${}^{f}$	196/194 ${}^{f}$
$\nu_{10}$	751.1(1)	747	743	753	748	759	762
$\nu_{11}$	n.a.	580	580	581	581	599	594
$\nu_{12}$	n.a.	235	233	236	233	238	237
Max Pos.	−	3.1	8.3	4.0	5.8	8.3	38.5
Max Neg.	−	−3.7	−8.1	−4.4	−2.6	−13.4	−20.9
MD	−	−0.4	1.0	0.4	1.7	0.9	4.1
MAD	−	1.4	3.4	1.5	2.6	4.1	12.0

*^a^* Harmonic frequencies at CCSD(T)/cc-pV5Z level, cubic and quartic force constants at rev-DSDPBEP86/juncc-pV(T+*d*)Z level. *^b^* Harmonic frequencies at CCSD(T)/cc-pV5Z level, cubic and quartic force constants at PW6B95/jul-cc-pV(D + *d*)Z level. *^c^* Harmonic frequencies from cheap composite scheme, cubic and quartic force constants at rev-DSDPBEP86/jun-cc-pV(T+*d*)Z level. *^d^* Harmonic frequencies from cheap composite scheme, cubic and quartic force constants at PW6B95/jul-cc-pV(D + *d*)Z level. *^e^* *v*_3_/*v*_10_ + *v*_11_ frequencies. The two bands are coupled by a Fermi type 2 resonance with equal mixing of the *v*_3_ = 1 and *v*_10_ = *v*_11_ = 1 levels. f ^35^Cl/^37^Cl.

**Table 9:** Theoretical anharmonic infrared intensities (km mol${}^{-1}$) for ${}^{35}$ClHC=CF${}_{2}$ fundamental vibrations.

Mode	CC5Z:rDSD ${}^{a}$	CC5Z:PW6 ${}^{b}$	ChS:rDSD ${}^{c}$	ChS:PW6 ${}^{d}$	rDSD	PW6
$\nu_{1}$	12.07	11.66	12.00	10.52	13.35	15.06
$\nu_{2}$	142.05	144.49	132.46	133.64	151.60	161.76
$\nu_{3}$	84.06	82.18	60.43/45.43 ^e^	81.85	93.26	110.61
$\nu_{4}$	115.04	120.49	111.17	116.09	126.46	122.99
$\nu_{5}$	84.26	78.91	99.33	97.74	110.50	119.70
$\nu_{6}$	8.78	8.69	8.52	8.43	8.43	8.61
$\nu_{7}$	3.13	3.15	2.97	2.99	2.89	2.72
$\nu_{8}$	1.43	1.41	1.41	1.40	1.30	1.48
$\nu_{9}$	1.93	1.91	1.88	1.86	1.92	1.99
$\nu_{10}$	34.01	34.39	34.58	34.96	35.93	36.64
$\nu_{11}$	0.86	0.96	0.85	0.95	0.49	0.43
$\nu_{12}$	0.66	0.57	0.63	0.54	0.57	0.65

*^a^* Harmonic intensities at CCSD(T)/cc-pV5Z level augmented by anharmonic contributions at rev- DSDPBEP86/jun-cc-pV(T+*d*)Z level. *^b^* Harmonic intensities at CCSD(T)/cc-pV5Z level augmented by anharmonic contributions at PW6B95/jul-cc-pV(D + *d*)Z level. *^c^* Harmonic intensities from cheap composite scheme augmented by anharmonic contributions at rev-DSDPBEP86/jun-cc-pV(T+*d*)Z level. *^d^* Harmonic intensities from cheap composite scheme augmented by anharmonic contributions at PW6B95/jul-cc-pV(D + *d*)Z level. *^e^* *v*_3_/*v*_10_ + *v*_11_. The two bands are coupled by a Fermi type 2 resonance with equal mixing of the *v*_3_ = 1 and *v*_10_ = *v*_11_ = 1 levels.

**Table 10:** Vibrational assignment of ClHC=CF${}_{2}$ and comparison to theoretical wavenumbers (cm${}^{-1}$).

Band	Exp.	CC5Z:rDSD ${}^{a}$	ChS:rDSD ${}^{b}$	Band	Exp.	CC5Z:rDSD ${}^{a}$	ChS:rDSD ${}^{b}$
$\nu_{8}$	431.8(3)	431	431	$\nu_{2}+\nu_{12}$	1980.6(3)	1983	1986
$2\nu_{12}$	473.5(3)	472	473	$\nu_{4}+\nu_{5}$	2169.3(1)	2167	2165
$\nu_{7}$	578.0(1)/577.4(1) ${}^{c}$	577	578/577 ${}^{c}$	$\nu_{3}+\nu_{5}$	2314.5(5)	2306	2299
$\nu_{5}-\nu_{12}$	736.2	735	736	$\nu_{2}+\nu_{7}$	2323.6(5)	2323	2326
$\nu_{9}+\nu_{10}-\nu_{9}$	747.9(5)	745	753	$2\nu_{4}$	2394.7(1)	2392	3286
$\nu_{10}$	751.1(1)	747	753	$\nu_{5}+2\nu_{9}$	2486.9(5)	2486	2479
$\nu_{11}+\nu_{12}$	813.4(5)	815	816	$\nu_{3}+\nu_{4}$ ^*e*^	2522.8(1)	2531	2519
$\nu_{6}$	844.9(1)/842.8(5) ${}^{c}$	843	845/847 ${}^{c}$	$\nu_{2}+\nu_{6}$	2599.2(5)	2596	2599
$\nu_{5}$	971.5(1)/970.2(1) ${}^{c}$	970	972/970 ${}^{c}$	$2\nu_{3}$ ${}^{f}$	2663.7(1)	2676	2659
$\nu_{10}+\nu_{12}$	987.8(3)	985	990	$\nu_{2}+\nu_{5}$	2712.8(1)	2713	2716
$\nu_{7}+\nu_{8}$	1007.5(3)	1008	1010	$\nu_{2}+\nu_{4}$	2938.2(3)	2939	2938
$\nu_{6}+\nu_{9}$	1038.4(3)	1037	1038	$\nu_{2}+\nu_{3}$	3074.9(5)	3079	3073
$\nu_{6}+\nu_{12}$	1079.1(3)	1078	1080	$\nu_{1}$	3135.9(3)	3135	3139
$2\nu_{7}$	1153.1(3)/1152.2(3) ${}^{c}$	1154	1156/1155 ${}^{c}$	$\nu_{1}+\nu_{9}$	3325.9(5)	3330	3334
$\nu_{5}+\nu_{9}$	1166.4(3)	1165	1166	$2\nu_{2}$	3482.7(1)	3484	3489
$\nu_{4}$	1200.7(1)	1200	1196	$\nu_{1}+\nu_{7}$	3711.9(5)	3712	3717
$\nu_{6}+\nu_{8}$	1273.7(3)	1271	1273	$\nu_{1}+\nu_{6}$	3972.1(5)	3978	3982
$\nu_{10}+\nu_{11}$	1324.9(3)	1325	1326/1341 ${}^{d}$	$\nu_{1}+\nu_{5}$	4099.8(3)	4104	4109
$\nu_{3}$	1341.7(3)	1342	1326/1341 ${}^{d}$	$\nu_{2}+\nu_{3}+\nu_{4}$	4261.7(5)	4259	4248
$\nu_{5}+\nu_{8}$	1399.5(3)	1400	1402	$\nu_{1}+\nu_{4}$	4327.8(3)	4328	4328
$2\nu_{10}$	1498.2(1)	1490	1501	$\nu_{1}+\nu_{6}+\nu_{8}$	4399.4(5)	4404	4411
$\nu_{5}+\nu_{7}$	1540.1(5)	1545	1547	$\nu_{1}+\nu_{3}$	4471.1(3)	4474	4468
$\nu_{3}+\nu_{12}$	1576.4(1)	1577	1568	$\nu_{1}+\nu_{2}$	4884.8(5)	4891	4869
$2\nu_{6}$	1683.1(1)/1677.5(5) ${}^{c}$	1680	1682/1676 ${}^{c}$	$\nu_{1}+\nu_{3}+\nu_{4}$	5654.6(5)	5657	5648
$\nu_{2}$	1747.5(1)	1749	1751	$\nu_{1}+\nu_{2}+\nu_{4}$	6058.9(5)	6074	6075
$\nu_{4}+\nu_{7}$	1777.5(1)	1776	1774	$2\nu_{1}$	6150.1(5)	6157	6166
$\nu_{5}+\nu_{6}$	1813.9(3)	1812	1814	$\nu_{1}+\nu_{2}+\nu_{3}$	6218.3(5)	6218	6214
$2\nu_{5}$	1939.8(3)/1937.3(5) ${}^{c}$	1938	1940/1938 ${}^{c}$				

*^a^* Harmonic frequencies at CCSD(T)/cc-pV5Z level, cubic and quartic force constants at rev-DSDPBEP86/juncc- pV(T+*d*)Z level. *^b^* Harmonic frequencies from cheap composite scheme, cubic and quartic force constants at rev-DSDPBEP86/jun-cc-pV(T+*d*)Z level. *^c^* ^35^Cl/^37^Cl. *^d^* *v*_3_/*v*_10_ + *v*_11_. The two bands are coupled by a Fermi type 2 resonance with equal mixing of the *v*_3_ = 1 and *v*_10_ = *v*_11_ = 1 levels. *^e^* Overlapped with *v*_4_ + *v*_10_ + *v*_11_ at 2523 cm^−1^ according to CC5Z:rDSD predictions. *^f^* Overlapped with *v*_3_ + *v*_10_ + *v*_11_ at 2668 cm^−1^ according to CC5Z:rDSD predictions.

**Table 11:** Experimental and theoretical integrated absorption cross sections ($10^{-19}$ cm molecule${}^{-1}$) of ClHC=CF${}_{2}$${}^{a}$.

Integration Limits/cm${}^{-1}$	Main Absorptions	Exp.	CC5Z:rDSD ${}^{b}$	ChS:rDSD ${}^{c}$
400–460	$\nu_{8}$	2.33(27)	2.37	2.46
530–620	$\nu_{7}$	6.503(80)	6.81	6.43
690–800	$\nu_{10}$	55.31(34)	56.56	57.52
800–890	$\nu_{6}$	16.05(10)	15.91	15.41
920–1080	$\nu_{5}$, $\nu_{10}+\nu_{12}$	182.0(16)	190.96	188.41
1090–1245	$\nu_{4}$, $2\nu_{11}$, $\nu_{5}+\nu_{9}$, $2\nu_{7}$	200.4(17)	204.11	209.95
1245–1450	$\nu_{3}$, $\nu_{6}+\nu_{8}$, $\nu_{10}+\nu_{11}$, $\nu_{5}+\nu_{8}$	193.4(13)	209.10	220.31
1450–1590	$2\nu_{10}$, $\nu_{5}+\nu_{7}$, $\nu_{3}+\nu_{12}$	11.24(28)	11.32	11.25
1590–1870	$\nu_{2}$, $\nu_{4}+\nu_{7}$, $\nu_{5}+\nu_{6}$, $2\nu_{6}$	263.4(28)	283.61	281.66
1870–1970	$2\nu_{5}$	1.767(34)	2.73	2.34
2090–2240	$\nu_{4}+\nu_{5}$	2.546(38)	3.95	3.40
2240–2360	$\nu_{3}+\nu_{5}$, $\nu_{2}+\nu_{7}$	2.69(10)	3.57	2.90
2360–2450	$2\nu_{4}$	2.459(80)	3.58	3.75
2480–2560	$\nu_{2}+\nu_{10}$, $\nu_{3}+\nu_{4}$	1.713(37)	4.75	1.92
2560–2800	$2\nu_{3}$, $\nu_{2}+\nu_{6}$, $\nu_{2}+\nu_{5}$	7.61(16)	19.87	17.47
2800–2990	$\nu_{2}+\nu_{4}$	1.32(15)	1.29	1.39
3020–3190	$\nu_{1}$, $\nu_{2}+\nu_{3}$	21.41(16)	22.67	22.04
3440–3500	$2\nu_{2}$	0.628(63)	0.84	0.85
4230–4350	$\nu_{1}+\nu_{4}$	0.94(20)	1.17	1.16
4430–4500	$\nu_{1}+\nu_{3}$	0.61(13)	0.86	0.72
4575–4690	$2\nu_{2}+\nu_{4}$	0.045(4)	0.019	0.018
4830–4920	$2\nu_{1}+\nu_{2}$	0.303(4)	0.35	0.37
5900–6290	2$\nu_{1}$	1.51(2)	1.71	1.68

*^a^* Values in parentheses are standard errors in the units of the last significant digits. *^b^* Harmonic frequencies and intensities at CCSD(T)/cc-pV5Z level, anharmonic contributions at rev-DSD-PBEP86-D3/jun-cc-pV(T+*d*)Z level. *^c^* Harmonic frequencies and intensities from ChS, anharmonic contributions at rev-DSD-PBEP86-D3/jun-ccpV(T+*d*)Z level.

## Supplementary Materials

The following are available online, Figure S1: Differences between theoretical and experimental wavenumbers for ${}^{35}$ClHC=CF${}_{2}$ fundamental transitions, Table S1: Theoretical equilibrium geometry, Table S2: Theoretical rotational spectroscopic parameters of ${}^{35}$ClHC=CF${}_{2}$, Table S3: Theoretical rotational spectroscopic parameters of ${}^{37}$ClHC=CF${}_{2}$, Table S4: Theoretical rotational spectroscopic parameters of ClHC=${}^{13}$CF${}_{2}$, Table S5: Theoretical rotational spectroscopic parameters of ClH${}^{13}$C=CF${}_{2}$, Table S6: Theoretical rotational spectroscopic parameters of ${}^{35}$ClDC=CF${}_{2}$, Table S7: Theoretical rotational spectroscopic parameters of ${}^{37}$ClDC=CF${}_{2}$, Table S8: Harmonic and anharmonic wavenumbers and intensities of ${}^{35}$ClHC=CF${}_{2}$ fundamental vibrations at B2PLYP/jun-cc-pV(T+*d*)Z level, Table S9: Harmonic and anharmonic wavenumbers and intensities of ${}^{35}$ClHC=CF${}_{2}$ fundamental vibrations at B3LYP/SNSD level, Table S10: Harmonic and anharmonic wavenumbers and intensities of ${}^{35}$ClHC=CF${}_{2}$ fundamental vibrations at rev-DSDPW6B95/jun-cc-pV(T+*d*)Z level, Table S11: Harmonic and anharmonic wavenumbers and intensities of ${}^{35}$ClHC=CF${}_{2}$ fundamental vibrations at PW6B95/jul-cc-pV(T+*d*)Z level, Table S12: Theoretical and experimental anharmonic constants of ${}^{35}$ClHC=CF${}_{2}$, Table S13: Comparison among the fundamentals (cm${}^{-1}$) of R1122 and similar halogenated ethenes.

## Sample Availability

Samples of the compounds are not available from the authors.
