# Supplementary material for: The Spectroscopic Characterization of Halogenated Pollutants through the Interplay between Theory and Experiment: Application to R1122
Source: Molecules. 2022 Jan 24;27(3):748. doi: 10.3390/molecules27030748 (PMC8839295; doi:10.3390/molecules27030748)
Supplement: Supplementary file 1 [file molecules-27-00748-s001.zip › molecules-1549704-supplementary.pdf]

Supplementary Materials: The spectroscopic characterization of halogenated pollutants through the interplay between theory and experiment: application to R1122

Andrea Pietropolli Charmet [1*], Giorgia Ceselin [2], Paolo Stoppa[1] and Nicola Tasinato [2,*]

1. List of supporting information available

Table S1. Theoretical equilibrium geometry.
Table S2. Theoretical rotational spectroscopic parameters of $^{35}ClHC{=}CF_2$.
Table S3. Theoretical rotational spectroscopic parameters of $^{37}ClHC{=}CF_2$.
Table S4. Theoretical rotational spectroscopic parameters of $ClHC{=}^{13}CF_2$.
Table S5. Theoretical rotational spectroscopic parameters of $ClH^{13}C{=}CF_2$.
Table S6. Theoretical rotational spectroscopic parameters of $^{35}ClDC{=}CF_2$.
Table S7. Theoretical rotational spectroscopic parameters of $^{37}ClDC{=}CF_2$.
Table S8. Harmonic and anharmonic wavenumbers and intensities of $^{35}ClHC{=}CF_2$ fundamental vibrations at B2PLYP/jun-cc-pV(T+*d*)Z level.
Table S9. Harmonic and anharmonic wavenumbers and intensities of $^{35}ClHC{=}CF_2$ fundamental vibrations at B3LYP/SNSD level.
Table S10. Harmonic and anharmonic wavenumbers and intensities of $^{35}ClHC{=}CF_2$ fundamental vibrations at rev-DSDPW6B95/jun-cc-pV(T+*d*)Z level.
Table S11. Harmonic and anharmonic wavenumbers and intensities of $^{35}ClHC{=}CF_2$ fundamental vibrations at PW6B95/jul-cc-pV(T+*d*)Z level.
Table S12. Theoretical and experimental anharmonic constants of $^{35}ClHC{=}CF_2$.
Table S13. Comparison among the fundamentals (cm^{-1}) of R1122 and similar halogenated ethenes.
Figure S1. Differences between theoretical and experimental wavenumbers for $^{35}ClHC{=}CF_2$ fundamental transitions.

Table S1: Theoretical equilibrium geometry of $ClHC{=}CF_2$.[a]

**parameter**	**CCSD(T)**[b]	**rDSD**[c]	**B2PLYP**[d]	**PW6B95**[e]	**B3LYP**[f]
r(C2−H1)	1.0764	1.0774	1.0748	1.0820	1.0802
r(C2=C3)	1.3292	1.3258	1.3230	1.3233	1.3266
r(C3−F4)	1.3177	1.3180	1.3198	1.3208	1.3254
r(C2−Cl5)	1.7246	1.7160	1.7172	1.7112	1.7365
r(C3−F6)	1.3114	1.3111	1.3127	1.3140	1.3177
α(C3C2H1)	120.32	120.29	120.33	120.46	120.95
α(C2C3F4)	123.06	123.19	123.20	123.30	123.03
α(C3C2Cl5)	121.90	121.94	122.13	122.03	122.19
α(C2C3F6)	125.73	125.77	125.83	125.81	125.92

[a] Bond lengths and angles in Å and °, respectively.
[b] CCSD(T)/cc-pVTZ
[c] rev-DSDPBEP86/jun-cc-pV(T+*d*)Z.
[d] B2PLYP/jun-cc-pV(T+*d*)Z.
[e] PW6B95/jul-cc-pV(D+*d*)Z.
[f] B3LYP/SNSD.

Table S2: Equilibrium (B_e^α) and ground state (B_0^α) rotational-, centrifugal distortion-, nuclear quadrupolar coupling constants of ^{35}ClHC=CF$_2$.[a]

	**PW6B95**[b]	**rDSD**[c]	**B2PLYP**[d]	**B3LYP**[e]	**Exp.**[f]
A_e	10693.117	10714.792	10705.289	10607.437	-
B_e	2288.825	2285.138	2281.690	2250.482	-
C_e	1885.275	1882.476	1880.808	1856.585	-
A_0	10637.626	10656.782	10647.339	10552.604	10710.73661(64)
B_0	2282.050	2277.883	2274.375	2243.407	2297.18720(14)
C_0	1877.510	1875.352	1872.684	1848.760	1890.14644(15)
Δ_J	0.355	0.339	0.338	0.330	0.348727(26)
Δ_{JK}	4.44	4.08	4.06	3.99	4.07532(51)
Δ_K	7.61	7.69	7.75	7.80	7.8803(52)
δ_J	0.0604	0.0579	0.0577	0.0563	0.059845(8)
δ_k	2.73	2.54	2.54	2.50	2.6008(14)
$\Phi_J \times 10^5$	7.63	6.03	6.15	6.24	-
$\Phi_{JK} \times 10^3$	5.62	4.01	4.18	4.27	-
Φ_{KJ}	-0.0355	-0.0308	-0.0305	-0.0299	-0.0278(24)
Φ_K	0.0607	0.0558	0.0554	0.0549	0.067(15)
$\phi_J \times 10^5$	2.00	1.73	1.75	1.76	-
$\phi_{JK} \times 10^3$	2.65	1.91	2.00	2.05	2.25(39)
Φ_K	0.103	0.0876	0.0879	0.0872	-
χ_{aa}	-51.0	-51.8	-52.4	-55.3	-54.7502(91)
χ_{bb}	17.7	17.4	18.1	18.9	18.0921(88)
χ_{cc}	33.3	34.3	34.3	36.4	36.658(10)
$\lvert\chi_{ab}\rvert$	43.0	43.5	-43.8	-45.4	46.62(31)

[a] Rotational parameters within the Watson's A-reduction Hamiltonian in the I^r representation. Rotational and nuclear quadrupolar coupling constants in MHz; centrifugal distortion parameters in kHz.
[b] PW6B95/jul-cc-pV(D+*d*)Z.
[c] rev-DSDPBEP86/jun-cc-pV(T+*d*)Z.
[d] B2PLYP/jun-cc-pV(T+*d*)Z.
[e] B3LYP/SNSD.
[f] Rotational- and centrifugal distortion constants from Ref. [1]; nuclear quadrupolar coupling constants from Ref. [2].

Table S3: Equilibrium (B_e^α) and ground state (B_0^α) rotational constants, centrifugal distortion-, nuclear quadrupolar coupling constants of $^{37}ClHC=CF_2$ and comparison to experimental values.[a]

	**ChS**[b]	**PW6B95**[c]	**rDSD**[d]	**B2PLYP**[e]	**B3LYP**[f]	**Exp.**[g]
A_e	10781.801	10693.117	10714.762	10705.289	10607.437	-
B_e	2240.245	2224.100	2220.473	2217.085	2186.626	-
C_e	1854.845	1841.145	1839.317	1836.708	1812.905	-
A_0	10723.791	10637.626	10656.752	10647.279	10552.605	10710.7013(16)
B_0	2233.230	2217.535	2213.458	2210.040	2179.821	2232.28022(67)
C_0	1846.991	1833.621	1831.462	1828.824	1805.350	1845.97825(49)
Δ_J	0.336	0.341	0.326	0.324	0.317	0.307(12)
Δ_{JK}	4.03	4.29	3.94	3.92	3.85	3.66(11)
Δ_K	7.99	7.78	7.84	7.90	7.95	8.62(15)
δ_J	0.0560	0.0567	0.0543	0.0541	0.0528	0.0575(11)
δ_k	2.51	2.64	2.45	2.45	2.41	2.03(13)
$\Phi_J \times 10^5$	5.95	7.20	5.66	5.78	5.86	-
$\Phi_{JK} \times 10^3$	2.75	5.26	3.74	3.90	3.99	-
Φ_{KJ}	-0.0228	-0.0343	-0.0298	-0.0295	-0.0288	-
Φ_K	0.0358	0.0599	0.0551	0.0547	0.0542	-
$\phi_J \times 10^5$	1.88	1.85	1.59	1.61	1.62	-
$\phi_{JK} \times 10^3$	1.34	2.47	1.77	1.86	1.91	
Φ_K	0.061	0.101	0.086	0.087	0.086	-
χ_{aa}	-43.28	-40.2	-40.8	-41.3	-43.6	-43.2687(56)
χ_{bb}	14.33	13.9	13.7	14.3	14.9	14.3688(66)
χ_{cc}	28.95	26.3	27.1	27.1	28.7	28.8999(63)
$\|\chi_{ab}\|$	36.63	33.8	34.3	34.6	35.9	36.77(50)

[a] Rotational parameters within the Watson's A-reduction Hamiltonian in the I^r representation. Rotational- and nuclear quadrupolar coupling constants in MHz; centrifugal distortion parameters in kHz
[b] Equilibrium rotational constants from ChS geometry; ground state rotational constants from ChS equilibrium rotational constants and rev-DSDPBEP86/jun-cc-pV(T+*d*)Z vibrational contributions; quartic centrifugal distortion constants from ChS; sextic centrifugal distortion parameters at CCSD(T)/cc-pVTZ level; nuclear quadrupolar coupling constants at a.e.-MP2/cc-pwCVTZ level.
[c] PW6B95/jul-cc-pV(D+*d*)Z.
[d] rev-DSDPBEP86/jun-cc-pV(T+*d*)Z.
[e] B2PLYP/jun-cc-pV(T+*d*)Z.
[f] B3LYP/SNSD.
[g] From Ref. [2].

Table S4: Equilibrium (B_e^{α}) and ground state (B_0^{α}) rotational constants, centrifugal distortion-, nuclear quadrupolar coupling constants of ^{35}ClHC=^{13}CF$_2$ and comparison to experimental values.[a]

	**ChS**[b]	**PW6B95**[c]	**rDSD**[d]	**Exp.**[e]
A_e	10780.705	10692.098	10713.773	-
B_e	2297.970	2281.451	2277.733	-
C_e	1894.211	1880.238	1878.380	-
A_0	10723.934	10637.656	10656.902	10710.8809(44)
B_0	2290.864	2274.825	2270.628	2289.8665(18)
C_0	1886.297	1872.624	1870.465	1885.19031(94)
Δ_J	0.329	0.352	0.337	0.403(48)
Δ_{JK}	3.79	4.44	4.07	5.60(61)
Δ_K	8.08	7.59	7.67	8.17
δ_J	0.0549	0.0599	0.0574	0.0567(39)
δ_k	2.39	2.73	2.54	2.418
$\Phi_J\ \times 10^5$	6.42	7.56	5.99	-
$\Phi_{JK}\ \times 10^3$	4.03	5.58	3.98	-
Φ_{KJ}	-0.0316	-0.0356	-0.0310	-
Φ_K	0.0555	0.0604	0.0556	-
$\phi_J\ \times 10^5$	1.84	1.98	1.72	-
$\phi_{JK}\ \times 10^3$	1.95	2.64	1.90	-
Φ_K	0.0872	0.103	0.0878	-
χ_{aa}	-54.93	-51.0	-51.8	-54.9162(87)
χ_{bb}	18.19	17.5	17.5	18.2683(87)
χ_{cc}	36.73	33.3	34.3	36.6479(92)
$\|\chi_{ab}\|$	46.24	42.9	43.4	45.96(39)

[a] Rotational parameters within the Watson's A-reduction Hamiltonian in the I^r representation. Rotational- and nuclear quadrupolar coupling constants in MHz; centrifugal distortion parameters in kHz
[b] Equilibrium rotational constants from ChS geometry; ground state rotational constants from ChS equilibrium rotational constants and rev-DSDPBEP86/jun-cc-pV(T+*d*)Z vibrational contributions; quartic centrifugal distortion constants from ChS; sextic centrifugal distortion parameters at CCSD(T)/cc-pVTZ level; nuclear quadrupolar coupling constants at a.e.-MP2/cc-pwCVTZ level.
[c] PW6B95/jul-cc-pV(D+*d*)Z.
[d] rev-DSDPBEP86/jun-cc-pV(T+*d*)Z.
[e] From Ref. [2].

Table S5: Equilibrium (B_e^α) and ground state (B_0^α) rotational constants, centrifugal distortion-, nuclear quadrupolar coupling constants of $^{35}ClH^{13}C{=}CF_2$ and comparison to experimental values.[a]

	**ChS**[b]	**PW6B95**[c]	**rDSD**[d]	**Exp.**[e]
A_e	10650.931	10565.286	10585.762	-
B_e	2304.658	2288.016	2284.359	-
C_e	1894.684	1880.718	1878.889	-
A_0	10592.981	10509.824	10527.812	10579.6945(51)
B_0	2297.523	2281.331	2277.224	2296.5052(21)
C_0	1886.680	1873.013	1870.885	1885.5650(10)
Δ_J	0.329	0.352	0.337	0.348(54)
Δ_{JK}	3.58	4.19	3.85	4.38(66)
Δ_K	7.60	7.10	7.20	8.9(11)
δ_J	0.0565	0.0617	0.0591	0.0711(39)
δ_k	2.35	2.67	2.49	2.418
$\Phi_J \times 10^5$	6.39	7.52	5.95	-
$\Phi_{JK} \times 10^3$	3.80	5.28	3.75	-
Φ_{KJ}	-0.0302	-0.0343	-0.0295	-
Φ_K	0.0511	0.0556	0.0509	-
$\phi_J \times 10^5$	1.91	2.07	1.79	-
$\phi_{JK} \times 10^3$	1.84	2.51	1.80	-
Φ_K	0.0825	0.0972	0.0832	-
χ_{aa}	-54.79	-50.9	-51.8	-54.7502(91)
χ_{bb}	18.05	17.6	17.4	18.0921(88)
χ_{cc}	36.73	33.3	34.3	36.658(10)
$\lvert\chi_{ab}\rvert$	46.3	43.0	43.4	46.62(31)

[a] Rotational parameters within the Watson's A-reduction Hamiltonian in the I^r representation. Rotational- and nuclear quadrupolar coupling constants in MHz; centrifugal distortion parameters in kHz

[b] Equilibrium rotational constants from ChS geometry; ground state rotational constants from ChS equilibrium rotational constants and rev-DSDPBEP86/jun-cc-pV(T+*d*)Z vibrational contributions; quartic centrifugal distortion constants from ChS; sextic centrifugal distortion parameters at CCSD(T)/cc-pVTZ level; nuclear quadrupolar coupling constants at a.e.-MP2/cc-pwCVTZ level.

[c] PW6B95/jul-cc-pV(D+*d*)Z.

[d] rev-DSDPBEP86/jun-cc-pV(T+*d*)Z.

[e] From Ref. [2].

Table S6: Equilibrium (B_e^α) and ground state (B_0^α) rotational constants, centrifugal distortion-, nuclear quadrupolar coupling constants of ^{35}ClDC=CF$_2$ and comparison to experimental values[a].

	**ChS**[b]	**PW6B95**[c]	**rDSD**[d]	**Exp.**[e]
A_e	10061.314	9980.661	10001.466	-
B_e	2304.786	2288.106	2284.478	-
C_e	1875.222	1861.381	1859.703	-
A_0	10006.392	9928.467	9946.544	9993.72067(32)
B_0	2296.812	2280.611	2276.504	2295.74786(22)
C_0	1866.737	1853.257	1851.218	1865.60899(19)
Δ_J	0.323	0.346	0.331	0.3318(26)
Δ_{JK}	2.898	3.38	3.11	3.182(27)
Δ_K	5.918	5.48	5.59	5.678(29)
δ_J	0.0595	0.0650	0.0622	0.06381(42)
δ_k	2.07	2.35	2.19	1.866(83)
$\Phi_J \times 10^5$	6.22	7.54	6.04	-
$\Phi_{JK} \times 10^3$	2.93	4.12	2.91	-
Φ_{KJ}	-0.0233	-0.0271	-0.0232	-
Φ_K	0.0361	0.0397	0.0363	-
$\phi_J \times 10^5$	2.00	2.26	1.95	-
$\phi_{JK} \times 10^3$	1.42	1.97	1.40	-
Φ_K	0.0614	0.0718	0.0617	-
χ_{aa}(Cl)	-54.72	-50.8	-51.6	-54.7054(10)
χ_{bb}(Cl)	18.02	17.5	17.3	18.0667(13)
χ_{cc}(Cl)	36.70	33.3	34.3	36.6387(11)
$\|\chi_{ab}\|$(Cl)	46.33	43.1	43.6	46.860(13)
χ_{aa}(D)	-0.089	-0.092	-0.088	-0.0857(27)
χ_{bb}(D)	0.200	0.204	0.197	0.1925(19)
χ_{cc}(D)	-0.111	-0.112	-0.109	-0.1069(14)
$\|\chi_{ab}\|$(D)	0.007	0.003	0.004	-

[a] Rotational parameters within the Watson's A-reduction Hamiltonian in the I^r representation. Rotational- and nuclear quadrupolar coupling constants in MHz; centrifugal distortion parameters in kHz

[b] Equilibrium rotational constants from ChS geometry; ground state rotational constants from ChS equilibrium rotational constants and rev-DSDPBEP86/jun-cc-pV(T+*d*)Z vibrational contributions; quartic centrifugal distortion constants from ChS; sextic centrifugal distortion parameters at CCSD(T)/cc-pVTZ level; nuclear quadrupolar coupling constants at a.e.-MP2/cc-pwCVTZ level.

[c] PW6B95/jul-cc-pV(D+*d*)Z.

[d] rev-DSDPBEP86/jun-cc-pV(T+*d*)Z.

[e] From Ref. [2].

Table S7: Equilibrium (B_e^α) and ground state (B_0^α) rotational constants, centrifugal distortion-, nuclear quadrupolar coupling constants of ^{37}ClDC=CF$_2$.[a]

	**ChS**[b]	**PW6B95**[c]	**rDSD**[c]	**Exp.**[d]
A_e	10061.033	9980.391	10001.166	-
B_e	2239.781	2223.621	2220.023	-
C_e	1831.953	1818.451	1816.742	-
A_0	10006.201	9928.167	9946.334	9993.40899(37)
B_0	2232.077	2216.336	2212.318	2231.06858(11)
C_0	1823.739	1810.567	1808.528	1822.654138(90)
Δ_J	0.328	0.332	0.318	0.3194(21)
Δ_{JK}	3.08	3.27	3.01	3.000(18)
Δ_K	5.80	5.61	5.70	5.728(61)
δ_J	0.0602	0.0611	0.0584	0.06091(41)
δ_k	2.165	2.27	2.12	1.866
$\Phi_J \times 10^5$	5.96	7.10	5.65	-
$\Phi_{JK} \times 10^3$	2.75	3.86	2.72	-
Φ_{KJ}	-0.0228	-0.0263	-0.0225	-
Φ_K	0.0358	0.0391	0.0358	-
$\phi_J \times 10^5$	1.88	2.09	1.79	-
$\phi_{JK} \times 10^3$	1.34	1.84	1.30	-
Φ_K	0.0606	0.0710	0.0610	-
χ_{aa}(Cl)	-43.17	-40.1	-40.7	-43.1558(11)
χ_{bb}(Cl)	14.24	13.9	13.7	14.2807(12)
χ_{cc}(Cl)	28.93	26.2	27.0	28.8751(11)
$\|\chi_{ab}\|$(Cl)	36.49	33.9	34.3	36.898(12)
χ_{aa}(D)	-0.089	-0.092	-0.087	-0.0852(27)
χ_{bb}(D)	0.200	0.204	0.197	0.1923(19)
χ_{cc}(D)	-0.111	-0.112	-0.109	-0.1071(11)
$\|\chi_{ab}\|$(D)	0.007	0.003	0.004	-

[a] Rotational parameters within the Watson's A-reduction Hamiltonian in the I^r representation. Rotational- and nuclear quadrupolar coupling constants in MHz; centrifugal distortion parameters in kHz

[b] Equilibrium rotational constants from ChS geometry; ground state rotational constants from ChS equilibrium rotational constants and rev-DSDPBEP86/jun-cc-pV(T+*d*)Z vibrational contributions; quartic centrifugal distortion constants from ChS; sextic centrifugal distortion parameters at CCSD(T)/cc-pVTZ level; nuclear quadrupolar coupling constants at a.e.-MP2/cc-pwCVTZ level.

[c] PW6B95/jul-cc-pV(D+*d*)Z.

[d] rev-DSDPBEP86/jun-cc-pV(T+*d*)Z.

[e] From Ref. [2].

Table S8: Harmonic and anharmonic wavenumbers (cm^{-1}) and intensities (km mol^{-1}) of $^{35}ClHC{=}CF_2$ fundamental vibrations at B2PLYP/jun-cc-pV(T+*d*)Z level of theory.

**Normal mode**	ω	I^{harm}	ν	I^{anharm}
1	3271	16.12	3145	13.75
2	1780	188.59	1743	156.90
3	1350	103.28	1317	89.82
4	1219	146.27	1195	140.05
5	987	125.0	969	118.42
6	850	10.14	838	7.87
7	581	2.86	576	2.80
8	435	1.30	430	1.21
9	196	1.88	195	1.93
10	778	37.60	761	36.30
11	604	0.39	592	0.36
12	239	0.56	236	0.59

Table S9: Harmonic and anharmonic wavenumbers (cm^{-1}) and intensities (km mol^{-1}) of $^{35}ClHC{=}CF_2$ fundamental vibrations at B3LYP/SNSD level of theory.

**Normal mode**	ω	I^{harm}	ν	I^{anharm}
1	3256	16.92	3128	14.52
2	1783	183.31	1749	135.06
3	1329	103.85	1298	90.10
4	1201	151.75	1177	143.85
5	976	133.46	958	121.87
6	837	13.25	825	12.42
7	572	2.89	567	2.84
8	429	1.20	423	1.11
9	195	2.01	194	2.02
10	764	38.68	747	37.27
11	592	0.39	582	0.36
12	235	0.64	233	0.66

Table S10: Harmonic and anharmonic wavenumbers (cm^{-1}) and intensities (km mol^{-1}) of $^{35}ClHC{=}CF_2$ fundamental vibrations at revDSD/jun-cc-pV(T+*d*)Z level of theory.

**Normal mode**	ω	I^{harm}	ν	I^{anharm}
1	3268	15.64	3143	13.35
2	1789	185.47	1752	151.60
3	1362	114.04	1328	93.26
4	1225	134.28	1201	126.46
5	993	119.75	974	110.50
6	856	9.053	844	8.43
7	583	2.96	577	2.89
8	437	1.39	431	1.30
9	196	1.89	195	1.92
10	776	37.21	759	35.93
11	606	0.52	595	0.49
12	240	0.54	238	0.57

Table S11: Harmonic and anharmonic wavenumbers (cm^{-1}) and intensities (km mol^{-1}) of $^{35}ClHC{=}CF_2$ fundamental vibrations at PW6B95-D3/jul-cc-pV(D+*d*)Z level of theory.

**Normal mode**	ω	I^{harm}	ν	I^{anharm}
1	3286	17.60	3161	15.06
2	1821	187.02	1786	161.76
3	1347	128.80	1321	110.61
4	1206	128.53	1192	122.99
5	991	129.69	975	119.70
6	858	10.26	846	8.61
7	575	2.78	570	2.72
8	434	1.57	430	1.48
9	188	1.98	196	1.99
10	781	37.68	762	36.64
11	604	0.36	594	0.43
12	241	0.70	237	0.65

Table S12: Theoretical and experimental anharmonic constants (cm^{-1}) of $^{35}ClHC{=}CF_2$[a]

i/j	1	2	3	4	5	6	7	8	9	10	11	12
1	-56.6<br>-60.8(6)											
2	5.8<br>1.4(5)	-7.3<br>-6.2(1)										
3	-2.9<br>-6.5(6)	-11.9<br>-14.3(4)	-3.8<br>-9.9(3)									
4	-6.9<br>-8.8(3)	-9.8<br>-10.0(3)	-10.3<br>-19.6(4)	-3.6<br>-3.4(1)								
5	-2.2<br>-7.6(2)	-6.8<br>-6.2(6)	-5.9<br>1.3(3)	-3.1<br>-2.9(6)	-1.6<br>-1.6(3)							
6	-2.0<br>-8.7(5)	3.0<br>6.8(3)	-2.7	-1.5	-2.2<br>-2.5(6)	-3.5<br>-3.4(1)						
7	-0.1<br>-2.0(5)	-3.2<br>-1.9(3)	-5.0	-0.8<br>-1.2(5)	-2.9<br>-9.4(1)	-0.3	-0.1<br>-1.5(3)					
8	0.3<br>-1.5(5)	-2.3	-1.6	-2.0	-1.0<br>-3.8(4)	-3.3<br>-3.0(4)	0.3<br>-2.3(3)	0.4				
9	-0.2	-1.1	-1.7	1.3	-0.6	-1.4	0.1	-0.1	0.4			
10	-15.5	-7.8	3.3	1.5	-4.2	-1.7	0.0	0.2	0.3<br>-3.2(3)	-2.4<br>-2.0(1)		
11	-1.3	-9.1	-1.5	-2.4	-2.4	-0.5	0.0	-0.3	-0.1	-2.7	-0.3	
12	-0.1	-1.6<br>-0.6(5)	-0.5	-0.4<br>-1.1(5)	-3.6	-0.3	0.5	-3.5<br>1.4(5)	0.4	2.6<br>1.4(6)	-0.5	0.6

[a] x_{ij} anharmonic constants from deperturbed CC5Z:rDSD hybrid force field (top entry) and determined experimentally (bottom entry). $x_{ij} = x_{ji}$.

Table S13: Comparison among the fundamentals (cm^{-1}) of R1122 and analogous halogenated ethenes.

**Fundamental**[a]	**ClHC=CH$_2$**[b]	*trans*-**ClFE**[c]	*cis*-**ClFE**[d]	**R1122**[e]	**R1113**[f]	**FHC=CH$_2$**[g]
ν_1	3129	3103	3114	3135.9	1800.6	3140.7
ν_2	3090	3094	3102	1747.5	1334.4	3094.5
ν_3	3040	1647	1661	1341.7	1216.2	3062.1
ν_4	1614	1296	1335	1200.7	1059.7	1655.6
ν_5	1370.03	1218	1232	971.5	691.4	1379.5
ν_6	1280.82	1127	1062	844.9	516.2	1305.2
ν_7	1030.91	876	812	578.0	462.3	1155.4
ν_8	720.21	447	656	431.8	338	927.8
ν_9	395	270	200	195[h]	188	482.9
ν_{10}	942.17	888	857	751.1	538.7	929.1
ν_{11}	896.57	784	735	580[h]	368	863.1
ν_{12}	618.57	270	442	235.3[i]	174	712.4

[a] All the data listed in the present Table refer to ^{35}Cl isotopologues.
[b] Experimental data taken from [3] and references therein.
[c] *trans*-**ClFE** stands for *trans*-ClHC=CHF; experimental low resolution infrared data taken from [4], the corresponding theoretical analysis was then reported by Cazzoli *et al.* [5].
[d] *cis*-**ClFE** stands for *cis*-ClHC=CHF; experimental low resolution infrared data taken from [4], the corresponding theoretical analysis was then reported by Gambi *et al.* [6].
[e] **R1122** experimental low resolution infrared data (present work).
[f] **R1113** stands for ClFC=CF$_2$; experimental low resolution infrared data taken from [7].
[g] Experimental data taken from [8].
[h] **R1122** best estimate theoretical prediction (present work).
[i] **R1122** estimated value from the analysis of infrared spectra (present work).

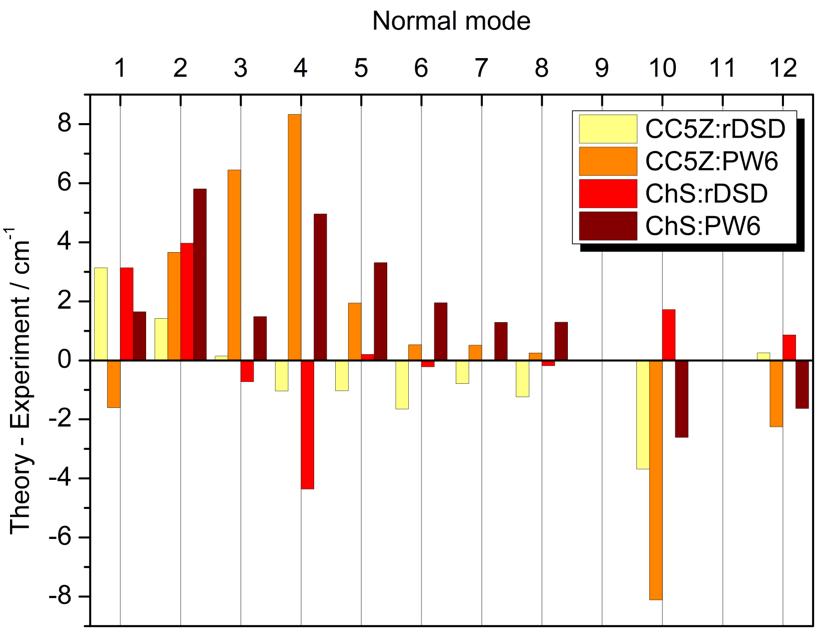

Figure S1. Difference between theoretical and experimental wavenumbers (cm^{-1} of $^{35}ClHC{=}CF_2$ fundamental frequencies of vibration obtained from different hybrid force fields).

References

1. Leal, L.A.; López, J.C.; Alonso, J.L.; Guarnieri, A. The Centimeter and Millimeter Microwave Spectrum of 1,1-Difluoro-2-Chloroethylene. *Z. Naturforsch.* **1993**, *48a*, 514–518. doi: doi:10.1515/zna-1993-0311.
2. Leung, H.O.; Marshall, M.D.; Messinger, J.P.; Knowlton, G.S.; Sundheim, K.M.; Cheung-Lau, J.C. The microwave spectra and molecular structure of 2-chloro-1,1-difluoroethene and its complex with the argon atom. *J. Mol. Spectrosc.* **2014**, *305*, 25–33. doi: 10.1016/j.jms.2014.09.011.
3. Demaison, J.; Møllendal, H.; Perrin, A.; Orphal, J.; Kwabia Tchana, F.; Rudolph, H.D.; Willaert, F. Microwave and high resolution infrared spectra of vinyl chloride, ab initio anharmonic force field and equilibrium structure. *J. Mol.Spectr.* **2005**, *232*, 174–185. doi: 10.1016/j.jms.2005.04.006.
4. Craig, N.C.; Lo, Y.S.; Piper, L.G.; Wheeler, J.C. Vibrational assignments and potential constants for cis- and trans-1-chloro-2-fluoroethylenes and their deuterated modifications. *J. Phys. Chem.* **1970**, *74*, 1712–1727. doi:10.1021/j100703a011.
5. Cazzoli, G.; Puzzarini, C.; Gambi, A. Trans-1-chloro-2-fluoroethylene: Microwave spectra and anharmonic force field. *J. Chem. Phys.* **2004**, *120*, 6495–6501. doi:10.1063/1.1667461.
6. Gambi, A.; Puzzarini, C.; Cazzoli, G.; Dore, L.; Palmieri, P. The anharmonic force field of cis-1-chloro-2-fluoroethylene. *Mol. Phys.* **2002**, *100*, 3535–3543. doi:10.1080/00268970210130155.
7. Tasinato, N.; Pietropolli Charmet, A.; Stoppa, P.; Giorgianni, S.; Gambi, A. Quantum-chemical ab initio investigation of the vibrational spectrum of halon 1113 and its anharmonic force field: A joint experimental and computational approach. *Chem. Phys.* **2012**, *397*, 55–64. doi: 10.1016/j.chemphys.2011.12.015.
8. Stoppa, P.; Pietropolli Charmet, A.; Tasinato, N.; Giorgianni, S.; Gambi, A. Infrared Spectra, Integrated Band Intensities, and Anharmonic Force Field of $H_2C{=}CHF$. *J. Phys. Chem. A* **2009**, *113*, 1497–1504. doi:10.1021/jp808556e.
